# Nitrous oxide is the main product during nitrate reduction by a novel lithoautotrophic iron(II)-oxidizing culture from an organic-rich paddy soil

**DOI:** 10.1128/aem.01262-24

**Published:** 2024-12-06

**Authors:** Hanna Grimm, Jennifer Lorenz, Daniel Straub, Prachi Joshi, Jeremiah Shuster, Christiane Zarfl, E. Marie Muehe, Andreas Kappler

**Affiliations:** 1 Geomicrobiology, Department of Geosciences, University of Tübingen 9188 , Tübingen, Germany; 2 Quantitative Biology Center (QBiC), University of Tübingen 9188 , Tübingen, Germany; 3 Tübingen Structural Microscopy Core Facility, University of Tübingen 9188 , Tübingen, Germany; 4 Environmental Systems Analysis, Department of Geosciences, University of Tübingen 9188 , Tübingen, Germany; 5 Plant Biogeochemistry, Department of Applied Microbial Ecology, Helmholtz Centre for Environmental Research - UFZ 545501 , Leipzig, Germany; 6 Plant Biogeochemistry, Department of Geosciences, University of Tübingen 9188 , Tübingen, Germany; 7 Cluster of Excellence: EXC 2124: Controlling Microbes to Fight Infection, Tübingen, Germany; Washington University in St. Louis, St. Louis, Missouri, USA

**Keywords:** culture HP, airborne microorganisms, greenhouse gas, arsenic, chemodenitrification, nitrogen, soil

## Abstract

Microbial nitrate reduction coupled to iron(II) oxidation (NRFeOx) occurs in paddy soils due to high levels of dissolved iron(II) and regular application of nitrogen fertilizer. However, to date, there is no lithoautotrophic NRFeOx isolate or enrichment culture available from this soil environment. Thus, resulting impacts on greenhouse gas emissions during nitrate reduction (i.e., nitrous oxide [N_2_O]) and on toxic metalloid (i.e., arsenic) mobility can hardly be investigated. We enriched a lithoautotrophic NRFeOx culture, culture HP (Huilongpu paddy, named after its origin), from a paddy soil (Huilongpu Town, China), which was dominated by *Gallionella* (71%). The culture reduced 0.45 to 0.63 mM nitrate and oxidized 1.76 to 2.31 mM iron(II) within 4 days leading to N_2_O as the main N-product (62%–88% N_2_O-N of total reduced NO_3_^−^-N). Nitrite was present as an intermediate at a maximum of 0.16 ± 0.1 mM. Cells were associated with, but mostly not encrusted by, poorly crystalline iron(III) minerals (ferrihydrite). Culture HP performed best below an iron(II) threshold of 2.5–3.5 mM and in a pH range of 6.50–7.05. In the presence of 100 µM arsenite, only 0%–18% of iron(II) was oxidized. Due to low iron(II) oxidation, arsenite was not immobilized. However, the proportion of N_2_O-N of total reduced NO_3_^−^-N decreased from 77% to 30%. Our results indicate that lithoautotrophic NRFeOx occurs even in organic-rich paddy soils, resulting in denitrification and subsequent N_2_O emissions. The obtained novel enrichment culture allows us to study the impact of lithoautotrophic NRFeOx on arsenic mobility and N_2_O emissions in paddy soils.

**IMPORTANCE:**

Paddy soils are naturally rich in iron(II) and regularly experience nitrogen inputs due to fertilization. Nitrogen fertilization increases nitrous oxide emissions as it is an intermediate product during nitrate reduction. Microorganisms can live using nitrate and iron(II) as electron acceptor and donor, respectively, but mostly require an organic co-substrate. By contrast, microorganisms that only rely on nitrate, iron(II), and CO_2_ could inhabit carbon-limited ecological niches. So far, no isolate or consortium of lithoautotrophic iron(II)-oxidizing, nitrate-reducing microorganisms has been obtained from paddy soil. Here, we describe a lithoautotrophic enrichment culture, dominated by a typical iron(II)-oxidizer (*Gallionella*), that oxidized iron(II) and reduced nitrate to nitrous oxide, negatively impacting greenhouse gas dynamics. High arsenic concentrations were toxic to the culture but decreased the proportion of nitrous oxide of the total reduced nitrate. Our results suggest that autotrophic nitrate reduction coupled with iron(II) oxidation is a relevant, previously overlooked process in paddy soils.

## INTRODUCTION

Nitrogen (N) fertilization is a common practice in agriculture, for example, in rice cultivation worldwide. Typically, N is applied in excess to paddy soils (as urea, ammonium-, or nitrate-based fertilizer), which results in poor nitrate (NO_3_^−^) use efficiencies and leaching of nitrate into groundwater (1–3). Nitrogen fertilization also influences the microbial processes in the paddy soil by providing a favorable electron acceptor under anoxic conditions that prevail under waterlogged conditions. Under anoxic conditions, nitrate can be removed microbially during dissimilatory nitrate reduction to ammonium (DNRA) or by denitrification. Denitrifying microorganisms are classified as (i) heterotrophs, which require an organic carbon substrate for energy generation, (ii) mixotrophs, which can use both organic carbon and inorganic compounds, and (iii) lithoautotrophs, which only use inorganic compounds such as hydrogen, reduced sulfur compounds, arsenite, or iron(II) (4). It was shown that different parameters such as pH, sulfide concentrations, type, and complexity of electron donors together with the ratio of organic carbon (OC) to N influence the likelihood of denitrification or DNRA (5). Typically, denitrification is the favored process for nitrate removal under lower OC/N ratios (6). In paddy soils, concentrations of dissolved or solid-phase iron(II) are naturally high due to anoxic conditions stimulating iron(III) reduction. Thus, nitrate reduction coupled with iron(II) oxidation (NRFeOx) is considered to play an important role in paddy soils (7).

Nitrate reduction is thermodynamically favored over sulfate reduction, iron(III) reduction, and methanogenesis, thereby limiting methane emissions and suppressing the reductive dissolution of iron(III) minerals. Given the high scavenging potential of toxic metalloids, like arsenic, by the iron(III) minerals formed by microbial iron(II) oxidation (8), NRFeOx can also limit the mobility of arsenic. Arsenic-contaminated groundwater is often used for paddy field irrigation (9, 10), thus, introducing arsenic to the soil and potentially resulting in accumulation within rice plants and grains (11). Arsenic is mainly present in its inorganic forms, arsenate and arsenite, with the latter being more toxic and mobile (12). However, iron(III) (oxyhydr)oxides that form during iron(II) oxidation have been shown to successfully sequester arsenate and arsenite during iron(II) oxidation (13), which play a key role in arsenic mobility. Depending on the speciation and concentration, arsenic can also pose toxic effects on microorganisms, including nitrate-reducing, iron(II)-oxidizing microorganisms (8).

Several studies have isolated or enriched heterotrophic or mixotrophic nitrate-reducing, iron(II)-oxidizing microorganisms from paddy soils and followed changes in the microbial community composition and diversity after the addition of nitrate, iron(II), or organic carbon (e.g., lactate, acetate) (14–17). Ratering and Schnell (7) postulated that the metabolic capacity for NRFeOx is widespread in paddy soils; however, lithoautotrophic microorganisms have not been isolated or enriched in the past from paddy soil. In these rather organic-rich environments (18), such microorganisms could inhabit ecological niches with low organic carbon and are generally considered to be better adapted to thrive under redox fluctuations where substrate availability may be scarce (19).

Three enrichment cultures have been described so far that unequivocally perform lithoautotrophic NRFeOx, originating from ditch sediments (i.e., culture KS, culture BP) and a limestone aquifer (i.e., culture AG) (20–22). *Gallionellaceae* sp. are dominant in all three cultures and share many common features such as genes encoding for iron(II) oxidation (e.g., *cyc2*), denitrification (e.g., *narGHI*, *nirK/S*, *norBC*), and carbon fixation (*rbcL*). The *Gallionellaceae* sp. in these three mixed cultures, however, are only capable of partial denitrification until NO or N_2_O, relying on a flanking community with other species such as *Rhodanobacter* sp. or *Bradyrhizobium* sp. for further nitrogen species removal (20, 23–25). Yet, 43%–96% and ~41% of the reduced nitrate accumulates as N_2_O in culture KS (26) and AG (21), respectively.

To investigate the important role of lithoautotrophic nitrate-reducing, iron(II)-oxidizing microorganisms for arsenic mobility and greenhouse gas emissions in paddy soils, we aimed to (i) obtain a model culture of lithoautotrophic nitrate-reducing, iron(II)-oxidizing microorganisms from paddy soil, (ii) identify microbial key players for NRFeOx using 16S rRNA gene amplicon sequencing, (iii) determine the extent of nitrate reduction, iron(II) oxidation, and N_2_O production, and (iv) compare growth conditions influencing the performance of the enrichment culture. Our novel enrichment culture offers a unique opportunity to further study NRFeOx in paddy soils, thereby enhancing our understanding of their impact on biogeochemical cycling, arsenic mobility, and climate change.

## RESULTS AND DISCUSSION

### Microbial community composition of the lithoautotrophic nitrate-reducing, iron(II)-oxidizing enrichment culture

**Fig 1:**
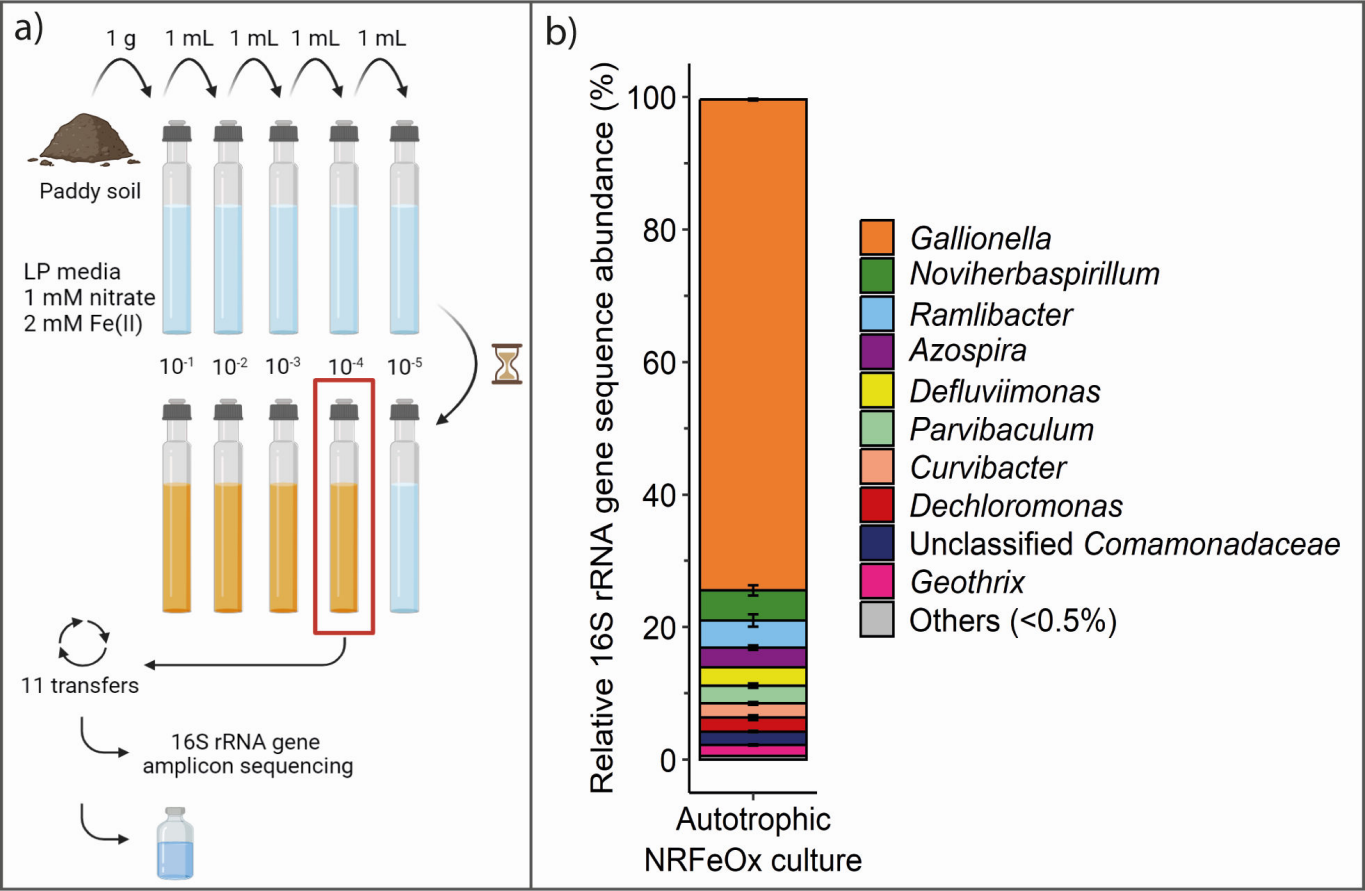
(**a**) Schematic of the enrichment and (**b**) microbial community composition at genus level of the lithoautotrophic nitrate-reducing, iron(II)-oxidizing enrichment culture HP from paddy soil. “Others” represent taxa with abundances below 0.5%. Error bars indicate the deviation from the mean of duplicate samples analyzed by 16S rRNA gene amplicon sequencing.

Lithoautotrophic nitrate-reducing, iron(II)-oxidizing microorganisms were enriched (Fig. 1a) by adding 1 g of paddy soil (Huilongpu Town, Hunan province, China) to 9 mL growth media containing 2 mM iron(II) and 1 mM nitrate (composition see Table S1). After observation of iron(II) oxidation and iron(III) mineral formation, indicated by a visual change from transparent to orange, 1 mL of the culture was transferred to 9 mL of fresh media. After 11 transfers under purely lithoautotrophic conditions by supplying 2 mM iron(II) as electron source and 1 mM nitrate as an electron acceptor, the microbial community composition of the NRFeOx culture was characterized by 16S rRNA gene amplicon sequencing (Fig. 1a). Two amplicon sequence variants (ASVs) classified as *Gallionella* (74.1±0.2%) dominated the microbial community of the successfully enriched NRFeOx culture HP (Huilongpu paddy, named after the origin of the soil, Fig. 1b). Members of *Gallionella* belong to the family *Gallionellaceae* of the order *Burkholderiales*. The more abundant *Gallionella* was present at 71.4% ± 0.3% and the other one at 2.7% ± 0.01% sharing 99.6% sequence identity (Fig. S1). Attempts to isolate the *Gallionella* species from the obtained NRFeOx culture involved different liquid or solid growth media (Table S2) with no success. The *Gallionella* did not grow under microoxic conditions in gradient tubes following the approach of Emerson and Floyd (27) (Table S2). In the native paddy soil, *Gallionella* accounted for 0.3% of the total microbial community (Table S3) and shared 94.4% sequence identity with the more abundant *Gallionella* in culture HP (Fig. S1).

In the three other known lithoautotrophic NRFeOx enrichment cultures (20–22), *Gallionellaceae* were also found to be the dominant family. Abundances of *Gallionellaceae* are similarly high in culture KS (96%), BP (71%–78%) and AG (49%) (20, 21, 23), compared to the novel enrichment culture HP described here. Previously, members of the family *Gallionellaceae* were characterized as autotrophic, neutrophilic, and microaerophilic iron(II)-oxidizers (28–30). However, *“Candidatus* Ferrigenium straubiae” sp.nov., *“Candidatus* Ferrigenium bremense” sp.nov., and *“Candidatus* Ferrigenium altingense” sp.nov. (in cultures KS, BP, and AG, respectively) perform, in tandem with their associated flanking communities, partial denitrification coupled to iron(II) oxidation and carbon fixation and are, thus, considered lithoautotrophic nitrate-reducing, iron(II)-oxidizing microorganisms. The enrichment culture in this study is most similar to culture AG based on the geochemical growth conditions (culture AG: 2 mM nitrate, 2 mM iron(II), this culture: 1 mM nitrate, 2 mM iron(II)). Maximum likelihood analysis revealed that the most dominant *Gallionella* species in culture HP was most similar to *“Ca*. ferrigenium altingense” sp. nov. from culture AG (Fig. S1), sharing 100% sequence identity (251 bp overlap). Even though the comparison of sequence similarities on species levels based on short-read 16S rRNA gene sequence analysis needs to be taken with caution, this similarity indicates that the potential for lithoautotrophic NRFeOx likely exists in many environments and with the appropriate culture conditions, microorganisms possessing this metabolic capability can be enriched and studied. Meta’omics revealed that “*Ca*. ferrigenium altingense” sp. nov. possesses genes for iron(II) oxidation, carbon fixation, and almost all genes involved in denitrification except for *nosZ* (25), which gives rise to the assumption that the *Gallionella* in culture HP has a similar metabolic potential.

The second most abundant ASV (4.5% ± 1.6%) in our lithoautotrophic NRFeOx culture HP belongs to the order *Burkholderiales* but was affiliated with the family *Oxalobacteraceae* and the genus *Noviherbaspirillum* (Fig. 1b). *Noviherbaspirillum* was also found in the flanking community of culture BP (9%–15%) (20). Meta’omics revealed that *Noviherbaspirillum* in culture BP has the potential to perform iron(II) oxidation, carbon fixation, and complete denitrification, likely playing an important role in NRFeOx.

Almost equally abundant like the *Noviherbaspirillum* in culture HP was *Ramlibacter* (4.1% ± 1.8%), belonging to the family of *Comamonadaceae* of the order *Burkholderiales* (Fig. 1b). *Ramlibacter* sp. in culture BP is considered to be involved in iron(II) oxidation and denitrification (*norB* and *nosZ* genes) (20). Other flanking community members in culture HP showed relative abundances at or below 3% and comprised members of the family *Rhodocyclaceae* (*Azospira,* 3.0% ± 0.7% and *Dechloromonas*, 2.1% ± 0.7%), which account for 0.14% of the native paddy soil microbial community (Table S3), *Comamonadaceae* (*Curvibacter*, 2.2% ± 0.5% and 1 ASV with unclassified genus, 2.0% ± 0.1%), making up 1.14% of the native paddy soil microbial community (Table S3), *Parvibaculaceae* (*Parvibaculum*, 2.7% ± 0.3%), *Rhodobacteraceae* (*Defluviimonas*, 2.8% ± 0.0%), and *Holophagaceae* (*Geothrix*, 1.7% ± 0.2%), accounting for 0.2% of the native paddy soil microbial community (Table S3; Fig. 1b). In culture KS, BP, or AG, *Azospira* was not present in the flanking community and was likely specific to culture HP and potentially to NRFeOx communities in paddy soils in general. The addition of iron(II), nitrate, and organic carbon (e.g., lactate or acetate) to paddy soils was shown to enrich *Azospira* in several studies (16, 17), suggesting that it might play an important role in hetero- or mixotrophic denitrification. Other flanking community members present in culture HP (Fig. 1b) (e.g., *Dechloromonas*, *Curvibacter,* and *Geothrix*) were also suggested before to be involved in Fe- and/or N-cycling (31–36).

### Nitrate removal and iron(II) oxidation under autotrophic growth conditions

**Fig 2:**
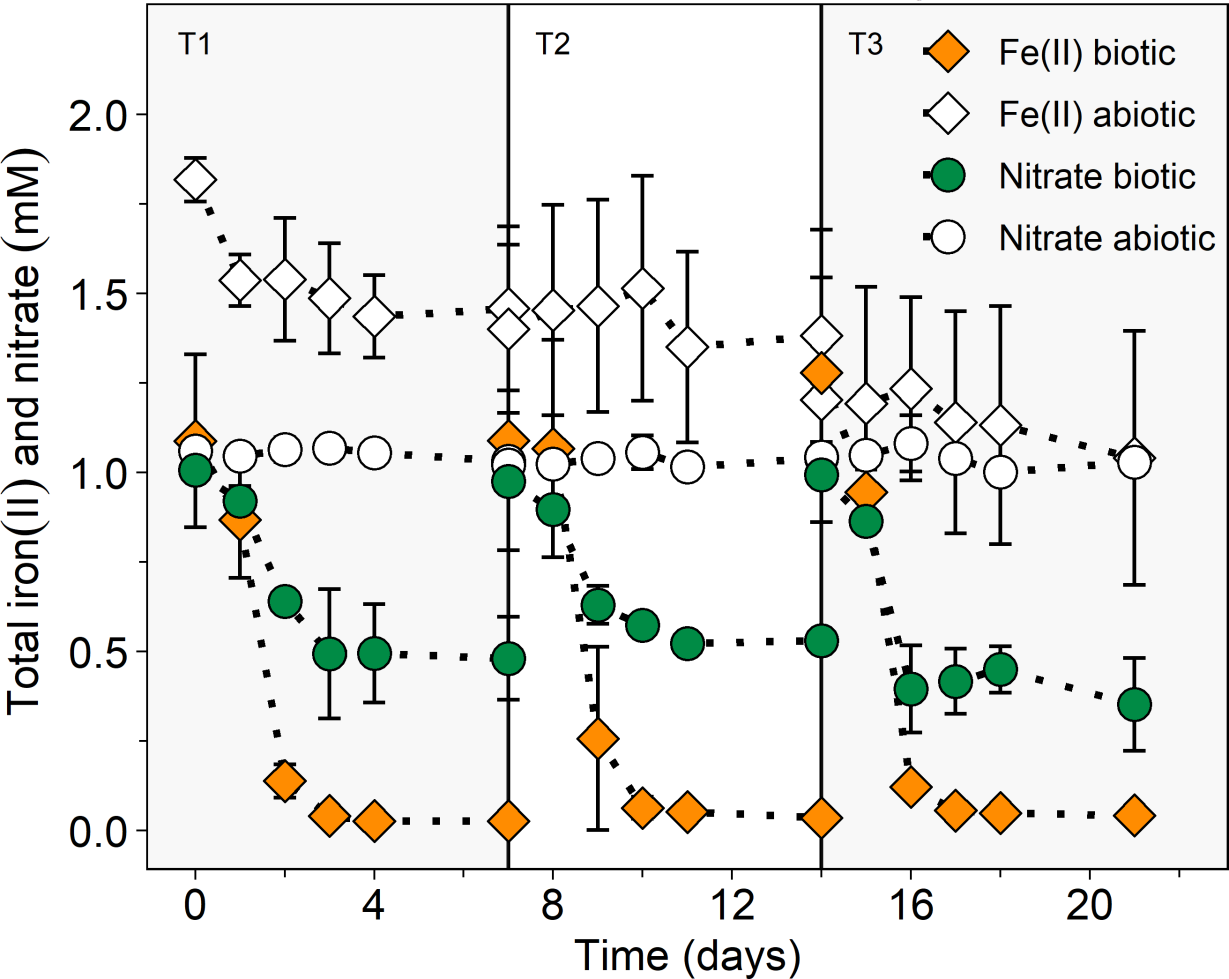
Total iron(II) (diamond) and nitrate (circle) concentrations over three consecutive transfers, each lasting over 7 days, of the lithoautotrophic nitrate-reducing, iron(II)-oxidizing enrichment culture HP from paddy soil. T1, T2, and T3 are visually separated by vertical lines and gray color and stand for transfer 1 (days 0–7), transfer 2 (days 7–14), and transfer 3 (days 14–21), respectively. Biotic treatments are represented as colored symbols and abiotic treatments are in white. Note that due to dilution by the microbial inoculum (10%, 2.5 mL), iron(II) concentrations were slightly lower in the biotic setups compared to abiotic setups. Mean ± standard deviation is shown of four replicates.

Nitrate reduction and iron(II) oxidation were monitored in detail over three consecutive transfers each lasting 7 days. In biotic treatments, 0.53 ± 0.12 mM, 0.45 ± 0.03 mM, and 0.64 ± 0.13 mM nitrate was reduced and 1.06 ± 0.24 mM, 1.05 ± 0.31 mM, and 1.24 ± 0.09 mM of iron(II) was oxidized within 7 days of incubation during transfer 1, 2, and 3, respectively (Fig. 2; Table S4). Because iron(II) precipitated on the inside surface of the glass serum bottles after the addition of FeCl_2_, measured iron(II) concentrations were lower than the theoretical 2 mM. Iron that was precipitated and could not be accounted for during the experiment was retrieved after the experiment by adding 1 M HCl (37). The ratio of iron(II) to iron(tot) decreased from 87.3% ± 28.7% to 1.6% ± 0.2% in transfer 1, from 88.3% ± 37.2% to 2.4% ± 0.4% in transfer 2, and from 90.9% ± 10.3% to 2.8% ± 0.2% in transfer 3. In culture AG, approximately 90% of the total iron(II) was oxidized (21), whereas 16%–26% iron(II) remained in culture KS (38) and 20% in culture BP (24), which were cultivated at higher iron(II) concentrations. 16S rRNA gene copy numbers increased, although not significantly (*P* = 0.12, Table S5, non-parametric Wilcoxon signed-rank test), from 3.4 × 10^3^ ± 1.6×10^3^ mL^−1^ to 4.3 × 10^5^ ± 2.5×10^5^ mL^−1^ and from 6.2 × 10^3^ ± 2.0×10^3^ mL^−1^ to 1.2 × 10^6^ ± 6.3 × 10^5^ mL^−1^ from day 0 till day 7 during transfers 2 and 3, respectively.

To investigate intermediate and final N-products during denitrification, aqueous nitrite concentrations and nitrous oxide emissions were quantified. Aqueous nitrite concentrations were generally low in biotic treatments, reaching a maximum of 0.08 ± 0.16 mM (day 3), 0.02 ± 0.02 mM (day 1), and 0.16 ± 0.1 mM (day 2) during transfers 1, 2, and 3, respectively (Fig. S2a). N_2_O production was determined by measuring gaseous concentrations and calculating dissolved concentrations based on Henry’s constant which revealed that 72.3% ± 19.4%, 88.5% ± 4.6%, and 62.4% ± 16.0% of the total reduced NO_3_^−^-N accumulated as N_2_O-N at the end (7 days) of transfers 1, 2, and 3, respectively (Table S4). Thus, N_2_O is the main product during lithoautotrophic NRFeOx in this culture. N_2_O also accumulated over time in culture KS and AG and represented 43%–96% (26) and ~41% (21) of the total reduced nitrate, respectively.

For calculations of the nitrate_reduced_ to iron(II)_oxidized_ ratio, the retrieved iron was considered and summed up with the final iron(tot) concentrations to obtain the true total iron concentrations. Considering that 90% of the total iron was present as iron(II) (with about 10% iron(III) stemming from the inoculum), we calculated that ratios of nitrate_reduced_ to iron(II)_oxidized_ were 0.23 ± 0.05, 0.23 ± 0.03, and 0.37 ± 0.08 in transfers 1, 2, and 3, respectively (Table S4). The higher ratio in transfer 3 was due to more reduced nitrate and less oxidized iron(II); however, the reason for this remains unidentified. Yet, the ratio in transfers 1 and 2 is close to the theoretical stoichiometry of 0.25, when assuming nitrate reduction to nitrous oxide (equation 1).


Eq. (1) $$2\;NO_{3}^{-}+8\;Fe^{2+}+19\;H_{2}O\;\to \;N_{2}O+8\;Fe{\left(OH\right)}_{3}+14\;H^{+}$$

However, the ratio of nitrate_reduced_ to iron(II)_oxidized_ should probably be slightly lower (ca. 0.21) considering that ~15% of the electrons from iron(II) oxidation are needed for carbon fixation (39) (equation 2).


Eq. (2) $$4\;Fe^{2+}+CO_{2}+11\;H_{2}O\to \;4\;Fe{\left(OH\right)}_{3}+CH_{2}O+8\;H^{+}$$

One explanation for a higher measured ratio than the theoretical ratio could be internally stored electrons or carbon leading to a greater nitrate reduction than theoretically possible under autotrophic conditions. Experiments without the addition of iron(II) showed a reduction of 0.05 ± 0.12 mM nitrate (Fig. S3). Considering this in the calculation of the nitrate_reduced_ to iron(II)_oxidized_ ratio, we achieved a ratio of 0.21, 0.20, and 0.34 for transfers 1, 2, and 3, respectively.

Without the addition of iron(II), 16S rRNA gene copy numbers increased from 2.5 × 10^4^ ± 1.1 × 10^4^ mL^−1^ (day 0) to 1.5 × 10^6^ ± 1.5 × 10^6^ mL^−1^ (day 7) (log2FC = 5.93, *P* = 0.25, Table S5, non-parametric Wilcoxon signed-rank test). The increase was similarly high as under standard conditions (log2FC = 6.65, 1.3 × 10^4^ ± 9.5×10^3^ mL^−1^ [day 0] to 1.3 × 10^6^ ± 7.0 × 10^5^ mL^−1^ [day 7], Table S6). Similar trends have been observed for the enrichment culture AG (21). When no iron(II) is supplied to the culture, biomass build-up could result from internally stored electrons or heterotrophic community members might be fueled by internally stored carbon. However, this underlines that 16S rRNA gene copy numbers alone have to be taken with caution when studying lithoautotrophic NRFeOx enrichment cultures or other cultures as well.

In abiotic treatments where no cells were added, nitrate concentrations stayed constant throughout the three transfers at around 1 mM (Fig. 2a). Iron(II) concentrations in the abiotic setups, however, decreased slowly within the 7 days of incubation (Fig. 2a). The decrease was strongest in the first transfer (1.82 ± 0.06 mM to 1.46 ± 0.23 mM) and less pronounced in the second and third transfers. This results from the precipitation of iron(II) minerals on the glass wall (37). The iron(II)/iron(tot) ratios stayed constant in the abiotic controls at 94.5% to 100%. Ammonium concentrations stemming from the growth media were constant throughout the three transfers at roughly 6 mM in biotic and abiotic treatments (Fig. S2b). The constant concentrations imply a minor role of DNRA or ammonium oxidation coupled to iron(III) reduction (Feammox).

Following the approach of Jakus et al. (21), 2 mM of iron(II) was re-spiked to culture HP after 7 days when all the initial iron(II) had been oxidized and nitrate had been reduced, to rule out that the lithoautotrophic NRFeOx enrichment culture HP grows on residual OC stemming from the MQ water. After re-spiking, iron(II) concentrations reached 1.71 ± 0.19 mM in the biotic treatments (Fig. S4). One day after the re-spike, 1.62 ± 0.19 mM of iron(II) was oxidized, corresponding to 95% of the re-spiked iron(II), and 0.38 ± 0.13 mM nitrate was reduced (less than 0.1 mM remaining nitrate) (Table S4). This corresponds to a molar ratio of nitrate_reduced_ to iron(II)_oxidized_ of 0.23 ± 0.08 (Table S4). Because precipitation of iron(II)-phosphate minerals at the glass wall did not occur in such a short timeframe, retrieved iron was not considered for the re-spike experiment. Due to very low nitrite concentrations, chemodenitrification likely plays a minor role, and iron(II) oxidation is assumed to be biotically catalyzed. 16S rRNA gene copy numbers were similar between day 0 and 7 days after the spike (day 0: 2.2 × 10^6^ ± 1.4 × 10^6^ mL^−1^, day 7: 1.3 × 10^6^ ± 6.1 × 10^6^ mL^−1^, *P* = 0.12, Table S5, non-parametric Wilcoxon signed-rank test). A 10 mM re-spike of iron(II) in culture KS also did not lead to a significant increase in cell number (38); however, a two-time spike of 2 mM iron(II) in culture AG led to an increase in cell numbers (21). In summary, the fast oxidation of iron(II) after the re-spike underlines that culture HP performs lithoautotrophic NRFeOx.

Continuous cultivation under lithoautotrophic conditions (ca. 20 transfers within 1 year) and different experimental setups and microbial and geochemical analyses confirm that the novel NRFeOx enrichment culture HP from a paddy soil simultaneously reduces nitrate and oxidizes iron(II) in the absence of any organic carbon source. The simultaneous increase in cell numbers over time (log2FC of 6.76 and 7.64 in transfers 2 and 3, respectively) as a measure for growth and the continuous, stable cultivation since 3 years (>60 transfers) under autotrophic conditions verifies that at least three out of four criteria are being met to prove true lithoautotrophic behavior (28). The fixation of labeled CO_2_ into biomass remains to be investigated. To the best of our knowledge, this is the first lithoautotrophic NRFeOx culture enriched from a soil environment, representing a rather organic-rich environment compared to the ditch sediment or aquifer material from which the lithoautotrophic NRFeOX cultures KS, AG, and BP have been enriched (20–22). Meta’omic analysis are needed in the future to determine the metabolic potential of culture HP and identify community members involved in iron(II) oxidation and certain steps of denitrification.

### Cell-mineral interactions

Microbial nitrate reduction coupled with iron(II) oxidation in the novel enrichment culture HP led to the formation of iron(III) (oxyhydr)oxide minerals. ^57^Fe-specific Moessbauer spectroscopy analysis identified a poorly crystalline iron(III) mineral phase (iron(III) oxyhydroxides) after 7 days of incubation (transfer 3) with hyperfine parameters similar to that of ferrihydrite (Table S7; Fig. S5).

**Fig 3:**
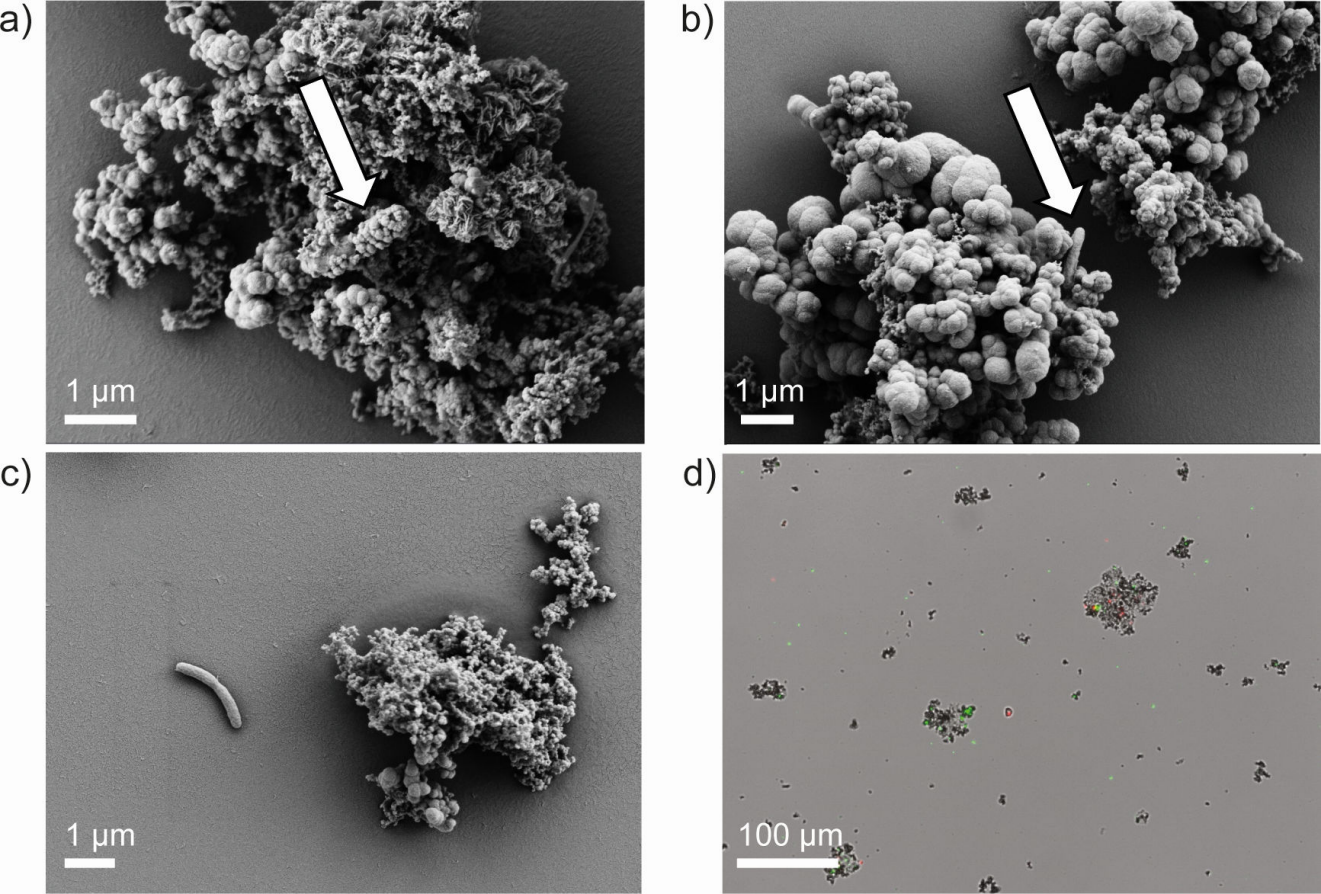
Scanning electron micrographs of the lithoautotrophic nitrate-reducing, iron(II)-oxidizing enrichment culture HP after 7 days (transfer 3) showing cell-mineral interactions of encrusted, mineral-associated (**a**), non-encrusted, mineral-associated (**b**), and non-encrusted (**c**) cells. Arrows point to cells. Overlay light micrograph of fluorescence and transmission light microscopic pictures (**d**). Cells were stained with the LIVE/DEAD stain (green, alive; red, dead).

Scanning electron microscopy (SEM) images revealed two dominant mineral structures, that is, mineral aggregates composed of either nanometer-scale particles (Fig. 3a) or nanometer- to micrometer-scale botryoidal-like particles (Fig. 3b). Energy dispersive spectroscopy analysis detected Fe, P, and O from these minerals (Fig. S6). Rod-shaped cells were closely associated with the newly formed iron(III) minerals (Fig. 3a). Only a few cells appeared encrusted by iron(III) minerals (Fig. 3b), whereas others were not encrusted or not in direct contact with iron(III) minerals (Fig. 3c). Light and fluorescence microscopy with LIVE/DEAD stain supported a close association of cells and iron(III) oxyhydroxides (Fig. 3d).

Autotrophic iron(II)-oxidizing microorganisms, i.e., nitrate-reducing, iron(II)-oxidizing microorganisms have been suggested to prevent cell encrustation during the precipitation of iron(III) minerals by excretion of iron(III)-complexing ligands, extracellular polymeric substances, modification of the cell surface charge or acidification of the microenvironment around the cell (40–43). In this study, SEM, fluorescence, and transmission light microscopy verified that most of the cells were free of encrustation irrespective of whether they were associated with the minerals or not. Only a few cells were partially or fully encrusted, which could resemble flanking community members that lack a prevention mechanism or dead cells that lost their ability to prevent encrustation (44, 45). However, Huang et al. (26) observed different degrees of encrustation in culture KS under varying nitrate-to-iron ratios with the degree of encrustation being lower when supplementing less iron. Yet, they did not find differences in mineral identity between different nitrate-to-iron ratios. The type of formed mineral was found to depend on growth conditions (46, 47) or metabolic processes (i.e., heterotroph vs. mixotroph vs. autotroph). Under heterotrophic or mixotrophic conditions, it was shown that abiotic iron(II) oxidation by nitrite (chemodenitrification) leads to the formation of goethite rather than poorly crystalline iron(III) (oxyhydr)oxides such as ferrihydrite (48–50). In the present study, the presence of a poorly crystalline iron(III) mineral phase (i.e., ferrihydrite) as identified by Moessbauer analysis and the low concentrations of nitrite point toward a minor role of chemodenitrification in this lithoautotrophic NRFeOx culture HP.

### Metabolic performance of the lithoautotrophic nitrate-reducing, iron(II)-oxidizing enrichment culture under various iron(II) concentrations and pH levels

**Fig 4:**
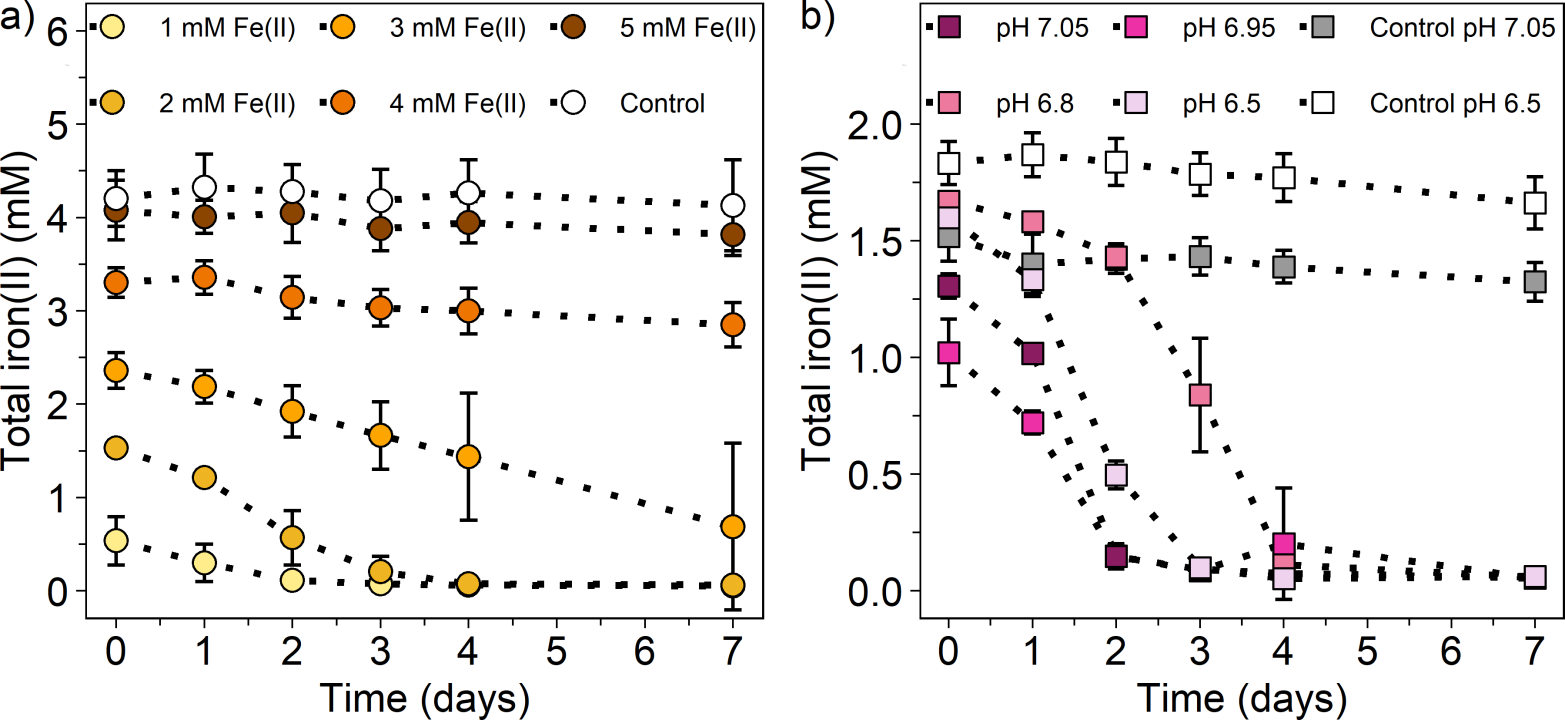
Total iron(II) concentrations for the lithoautotrophic nitrate-reducing, iron(II)-oxidizing enrichment culture HP from a paddy soil set up (**a**) with different iron(II) concentrations and (**b**) at different pH values over 7 days. Treatments in (**a**) were supplemented with 1 mM nitrate and different iron(II) concentrations, shown as circles with darker colors representing higher iron(II) concentrations. The control (white circles) represents abiotic conditions with 5 mM iron(II) and 1 mM nitrate. Treatments in (**b**) display different pH values as squares with darker colors representing higher pH values using 2 mM iron(II) and 1 mM nitrate. The controls represent abiotic conditions at pH 6.50 (white) and pH 7.05 (gray) using 2 mM iron(II) and 1 mM nitrate. Note that due to dilution by the microbial inoculum (10%, 2.5 mL), iron(II) concentrations were slightly lower in the biotic setups compared to abiotic setups. Mean ± standard deviation is shown of three replicates.

In paddy soils, concentrations of dissolved iron(II) can vary greatly due to changes in redox conditions (51). Thus, we investigated the performance of the lithoautotrophic NRFeOx enrichment culture HP in the presence of different iron(II) concentrations. When providing 1, 2, and 3 mM iron(II), 0.74 ± 0.27, 1.71 ± 0.03, and 1.88 ± 0.9 mM iron(II) was oxidized within 7 days, respectively (Fig. 4a; Table S4). With increasing iron(II) concentrations, a lower extent of iron(II) oxidation was observed (4 mM iron(II): 0.45 ± 0.29 mM, 5 mM iron(II): 0.29 ± 0.35 mM) (Fig. 4a; Table S4). At the same time, the extent of nitrate reduction was greatest for the 2 mM and 3 mM iron(II) setup, with 0.46 ± 0.16 and 0.41 ± 0.21 mM nitrate being reduced, respectively (Fig. S7a; Table S4). The extent of nitrate reduction was lower at iron(II) concentrations of 1 mM (0.12 ± 0.07 mM), 4 mM (0.12 ± 0.08 mM), or 5 mM (0.08 ± 0.04 mM) (Fig. S7a; Table S4). The ratio of nitrate_reduced_ to iron(II)_oxidized_ ranged between 0.16 and 0.29 (Table S4), being lowest in the 1 mM iron(II) treatment and highest in the 5 mM iron(II) treatment. These results highlight that our NRFeOx culture performs best when 2 mM iron(II) is supplied with relatively less nitrate being reduced and iron(II) being oxidized the higher or lower the concentration of iron(II) is. It also emphasizes that iron(II) concentrations can be crucial for the success of enriching or isolating lithoautotrophic nitrate-reducing, iron(II)-oxidizing microorganisms from different environments, likely due to toxicity effects of high concentrations of iron(II) (52). However, other enrichment cultures are likely less susceptible to higher iron(II) concentrations, especially culture KS and BP, that are routinely cultivated with 10 mM of iron(II). Culture AG grew slower with 3 mM of iron(II), but still oxidized almost all iron(II) (21). Yet, it still remains open where the upper and lower limit of iron(II) for microbial growth, nitrate reduction, and iron(II) oxidation of the other lithoautotrophic enrichment cultures is set.

Fluctuations in pH are also typically common in paddy soil due to flooding and drainage (53, 54); thus, we wanted to explore the pH range for enzymatic iron(II) oxidation by this enrichment culture. We found that the enrichment culture HP oxidized iron(II) from a pH of 6.50 to 7.05 without major differences in the extent but with differences in lag phases of iron(II) oxidation (Fig. 4b) or reduced nitrate (Fig. S7b). It has to be noted that the recovered initial iron(II) concentrations were slightly higher at lower pH values due to a larger extent of precipitation of iron(II) (at the glass wall) at higher pH values (55), which was also observed in the abiotic controls. Changes in pH between the beginning and the end of the experiment were minor (<0.1 pH unit, Table S8). Reduced nitrate ranged between 0.28 and 0.4 mM and oxidized iron(II) between 1.13 and 1.66 mM (Table S4). However, in all treatments, around 100% of iron(II) was oxidized. The ratio of nitrate_reduced_ to iron(II)_oxidized_ ranged between 0.22 and 0.25 (Table S4) and was well in line with the expected ratio of 0.25 or 0.21 when also considering biomass buildup. At a pH of 6.80, iron(II) oxidation and nitrate reduction were retarded by 1 day; however, at an even lower pH (pH 6.50), the culture HP behaved similarly to higher pH values. Different pH values could affect the contribution of chemodenitrification to iron(II) oxidation as shown by Zhu-Barker et al. (56). However, since nitrite was generally low and only observed for one treatment (pH 6.80) at day 1 (0.01 mM), we consider the contribution of chemodenitrification to iron(II) oxidation at all tested pH values as neglectable. Assuming a pH development during the rice growing season as modeled by Ding et al. (53) for paddy soils with an initial pH >6.5 (Table S9), where the pH slightly drops after flooding, stabilizes at pH 7 after around 30 days, and increases with decreasing moisture content, our results suggest that lithoautotrophic NRFeOx could occur throughout the rice-growing season. This indicates that the metabolic activity of these microorganisms is potentially sustained under the varying pH conditions typical of paddy soils.

### Metabolic performance of the lithoautotrophic nitrate-reducing, iron(II)-oxidizing enrichment culture after arsenite addition

In paddy soils, waterlogged conditions are responsible for a reducing environment where the highly toxic and mobile arsenite is the dominant arsenic species in the porewater (57–60). In studies using bacterial cultures or isolates and in paddy soil microcosm studies, similar arsenite concentrations or even higher concentrations [5,000 µM arsenate (61) and 500 µM arsenite (62)] were used and shown to not affect the metabolic capacity of the microorganisms (8, 61, 63–65). Thus, to allow comparison between previous studies on mixotrophic nitrate-reducing and phototrophic iron(II)-oxidizers (8), we supplemented 100 µM arsenite to culture HP, which allows us to better understand the limits and responses of the microbial community to arsenite toxicity, which would otherwise be more subtle or undetectable at lower concentrations.

We found that 13%–25% of nitrate was reduced when 100 µM arsenite was supplemented compared to 90%–98% without the presence of arsenite (Fig. S8a; Table S4). At the same time, 17.8% iron(II) was oxidized in the first transfer in the presence of arsenite and 0%–0.8% in transfers 2 and 3, which is lower compared to the setup without arsenite (62.5%–83.7% oxidized iron(II), Fig. S8b; Table S4). This points toward a toxic effect of arsenite on the lithoautotrophic NRFeOx enrichment culture HP. However, it remains open if the whole community in the culture or only specific community members were affected. Other iron(II)-oxidizing microorganisms, such as the mixotrophic strain BoFeN1, the lithoautotrophic culture KS, or phototrophic iron(II)-oxidizer *Rhodobacter ferrooxidans* strain SW2 were capable of thriving with a longer lag phase at the presence of 100 µM arsenite and even immobilized arsenite (initially: 50 µM arsenite) by newly formed iron(III) minerals (8). Thus, it remains to be investigated if arsenite still exerts toxicity effects under lower concentrations and if it can be successfully immobilized by iron(III) minerals formed during NRFeOx. Even though environmental concentrations of arsenite in the paddy soil are usually lower (58), our results suggest that the contribution of lithoautotrophic nitrate-reducing, iron(II)-oxidizing microorganisms in immobilizing arsenite in paddy soils might be lowered if initial concentrations of arsenite are as high as 100 µM, especially if the native microbial community is not adapted to high concentrations of arsenic in the paddy soil (here: 5 mg of 6 M HCl-extractable arsenic per kg soil, considerably low).

### Variability of nitrous oxide production under different growth conditions

**Fig 5:**
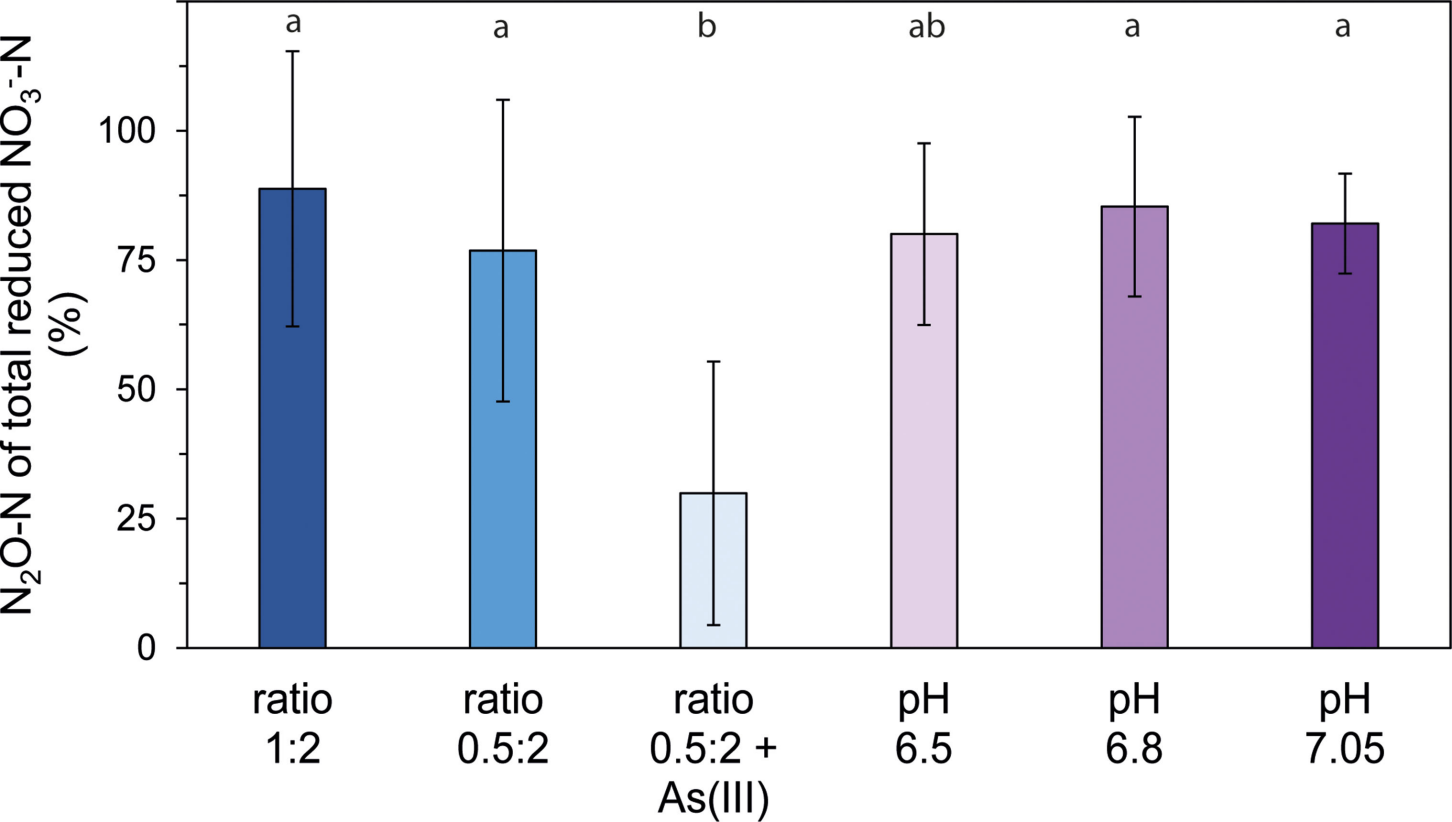
Relative N_2_O-N of total reduced NO_3_^−^-N of different experimental setups (different colors) of the lithoautotrophic nitrate-reducing, iron(II)-oxidizing enrichment culture HP from a paddy soil included different ratios of nitrate to iron(II), arsenite addition, and different pH values at standard conditions (nitrate to iron ratio of 1:2). Significant differences between treatments are indicated in small letters (significance level: *P* < 0.05, Table S10). Bars show the mean of replicates (ratio 1:2: *n* = 15, ratio 0.5:2: *n* = 9, ratio 0.5:2 + As(III): *n* = 9, pH 6.5: *n* = 3, pH 6.8: *n* = 3, pH 7.05: *n* = 3) and the error bars represent the standard deviation.

The percentages of N_2_O-N of the total reduced NO_3_^−^-N of different experimental setups were compared to identify differences in the extent of N_2_O production (Fig. 5; Table S10). We found that N_2_O production was significantly different between treatments (*P* < 0.01, Table S10a, non-parametric Kruskal-Wallis test). However, it was similar in treatments with different ratios of nitrate to iron(II) or different pH values. Under standard conditions (ratio N:Fe = 1:2, pH 6.95–7, *n* = 15), we calculated that on average 88.8% ± 26.6% of the reduced nitrate was converted to N_2_O; however, values ranged from 50% to 110% N_2_O-N of the total reduced NO_3_^−^-N. Due to low concentrations of nitrite, we postulate that N_2_O emissions are biologically derived and that chemodenitrification plays a minor role in the lithoautotrophic NRFeOx culture HP. Previously, N_2_O emissions in paddy soils have been attributed partly to chemodenitrification (66). Wang et al. (67) calculated, based on chemodenitrification rates from mixotrophic NRFeOx cultures, that chemodenitrification accounted for 6.8% to 67.6% of the total N_2_O emissions in two different paddy soils and postulated that the organic carbon content and iron(II) concentrations (determining the likelihood of DNRA or denitrification) are important for the contribution of chemodenitrification. However, our results suggest that lithoautotrophic NRFeOx leads almost exclusively to N_2_O emissions. Thus, it remains open if biologically derived N_2_O was previously underestimated in paddy soils. To better estimate the contribution of abiotic and biotic processes, the analysis of characteristic ^15^N and ^18^O fractionation patterns for chemodenitrification could be used (68) or kinetic modeling approaches similar to Jamieson et al. (69) and Liu et al. (50), including lithoautotrophic NRFeOx cultures, could be applied to disentangle the processes.

Growing culture HP under different pH values decreased the percentage of N_2_O-N of the total reduced NO_3_^−^-N only slightly to 85.3% ± 17.4% (pH 6.80, *n* = 3), 82% ± 9.7% (pH 7.05, *n* = 3) and 80% ± 17.6% (pH 6.50, *n* = 3). Thus, a potential inhibition of the *nosZ* gene due to lower pH values (70) can be excluded. Supplying culture HP with less nitrate (0.5 mM instead of 1 mM), which means that the electron acceptor is not in excess, also lowered the percentage of N_2_O-N of the total reduced NO_3_^−^-N to 76.8% ± 29.1% (ratio N:Fe = 0.5:2, pH 6.95–7, *n* = 9). In culture KS, N_2_O emissions were also found to depend on the ratio of nitrate to iron, with lower N_2_O under nitrate (electron acceptor) limitation (26). However, we only found a significant difference (*P* < 0.05, Table S10b, non-parametric Wilcoxon rank-sum test) when supplying 100 µM arsenite to culture HP, which lowered the percentage of N_2_O-N of the total reduced NO_3_^−^-N to 29.9% ± 25.5% (*n* = 9). With consecutive transfers of the culture on only nitrate and arsenite, less nitrate was reduced and mainly nitrite was the product of denitrification (Fig. S8c).

### Implications for nitrate reduction coupled to iron(II) oxidation in paddy soils

In paddy soils, several studies have emphasized the importance of iron(II)-oxidizing microorganisms, especially of *Gallionellaceae* (71–75). Watanabe et al. (73) estimated that *Gallionella*-related iron(II)-oxidizers contribute 4.6% to iron(II) oxidation in paddy soils, mainly due to microaerophilic iron(II) oxidation. *Ferrigenium kumadai* An22, a known microaerophilic iron(II)-oxidizer, has been isolated from paddy soil (76) and microorganisms of the order *Dechloromonas*, *Azospira*, *Zoogloea,* or *Pseudomonas* have been enriched in paddy soils after iron(II), nitrate, and organic carbon addition (14–17). However, to our knowledge, no study has focused before on lithoautotrophic NRFeOx in paddy soils, despite its important role in other environments (28, 39). The successful enrichment of a lithoautotrophic nitrate-reducing, iron(II)-oxidizing culture highlights that this metabolism was previously overlooked, yet is potential of relevance in paddy soils. We suggest that paddy soils provide an ideal environment for lithoautotrophic nitrate-reducing, iron(II)-oxidizing microorganisms that could inhabit ecological niches formed due to fluctuating redox conditions creating microenvironments influenced by nitrogen fertilization and naturally rich in iron(II) and CO_2_ (end product of organic matter decomposition). *Gallionellaceae* could be in close associations with roots, where oxygen concentrations vary depending on the growth stage of the plant (77), enabling it to switch from microaerophilic iron(II) oxidation to lithoautotrophic NRFeOx. (29) showed that *Gallionella* from a paddy soil rhizosphere possess *cyc2* for iron(II) oxidation, *nirK* for nitrite reductase, and *rbcL* for carbon fixation, giving rise to the assumption that *Gallionella* could perform lithoautotrophic NRFeOx, at least in interdependence with other microorganisms similar to culture BP and KS (20, 24). Whether this metabolic flexibility occurs in the environment remains to be investigated. Based on our results, *Gallionella* would require other microorganisms that are capable of N_2_O reduction. However, *Gallionella* and other lithoautotrophic nitrate-reducing, iron(II)-oxidizing microorganisms take up and sequester CO_2_, thereby reducing the CO_2_ concentration in the atmosphere (30), and in this way, could provide organic carbon for closely associated heterotrophic microorganisms, especially in times when organic carbon is limited. Further studies on lithoautotrophic NRFeOx in paddy soils, that is, with this novel culture HP, should focus on the metabolic potential of the dominant *Gallionella* and of the flanking community by meta‘omics analysis.

The successful enrichment of lithoautotrophic nitrate-reducing, iron(II)-oxidizing microorganisms from paddy soil addresses several unresolved research questions. Specifically, it allows us to investigate the culture’s capability to oxidize solid-phase iron(II), a process observed in other NRFeOx cultures or isolates (78–80). This could be particularly relevant in paddy soils rich in bioavailable and redox-sensitive iron(II) minerals (75, 81). In addition, it enables the study of the effects of other electron donors such as H_2_, sulfide, methane, or organic compounds, like short-chain fatty acids (originating from fermenting bacteria), soil-derived OC (dissolved OC in porewater), or root-derived OC (by rice plants) on N_2_O emissions and the microbial community composition. Ultimately, this novel lithoautotrophic NRFeOx enrichment culture HP provides a valuable basis for studying these processes in organic-rich environments.

## MATERIALS AND METHODS

### Field site, soil sampling, and soil characterization

Paddy soil samples were collected in September 2020 from a rice paddy field in Huilongpu Town, Hunan province, China (28°12′16″ N, 112°26′32″ E). The paddy soil is cultivated in a rice-rice cropping rotation and fertilized with 330 kg N ha^−1^ year^−1^ using urea. Soil was sampled from the upper 20 cm and stored at 4°C in the dark until further processing. After the removal of plant debris and larger gravel, basic soil properties were analyzed in triplicates, comprising analyses of soil texture, water content, pH, cation exchange capacity, total elemental content, total organic carbon and total N content, water-extractable organic carbon and inorganic N-species, and sequentially extractable iron and arsenic (1 M sodium acetate, 0.5 M HCl, and 6 M HCl). Detailed information is reported in the Appendix (Soil characterization, Table S9). Briefly, the paddy soil contains around 14 mg kg^−1^ and 5 mg kg^−1^ extractable iron and arsenic, respectively, and has a TOC content of 3.5%.

### Microbial enrichment

Microbial enrichments for lithoautotrophic nitrate-reducing, iron(II)-oxidizing microorganisms were set up in April 2021 by weighing 1 g of fresh paddy soil under sterile conditions into a sterile and anoxic Hungate tube. While continuously flushing the tube with N_2_:CO_2_ (90:10), 9 mL of anoxic, sterile modified low phosphate media (composition Table S1) supplemented with 1 mM nitrate (as KNO_3_) and 2 mM iron(II) (as FeCl_2_) was added and the tube was closed with a butyl stopper. By this, the electron donor, that is, nitrate, was present in excess (nitrate to iron ratio of 1:2). Afterwards, the Hungate tube was well mixed and a 10^−5^ dilution series into subsequent tubes was prepared by always transferring 1 mL into 9 mL of fresh growth media (82). The most positive dilution (based on color change from transparent to orange, indicating iron(II) oxidation) was always transferred into new Hungate tubes in a 10^−5^ dilution series as soon as color change appeared. This procedure was carried out over 11 transfers after which culture HP (Huilongpu paddy, named after the origin of the soil) was transferred into 25 mL serum bottles for further cultivation containing the same growth media and the same concentrations of iron(II) and nitrate. The culture HP was continuously transferred every 2–3 weeks and incubated at 25°C in the dark over roughly 10 months until culture HP was characterized.

### Experimental setups

After 11 transfers, the extent of nitrate reduction, iron(II) oxidation, and nitrous oxide production were followed over three consecutive transfers in four replicates (experimental setup illustrated in Fig. S9). For this, growth media was supplemented with 1 mM nitrate (as KNO_3_) and 2 mM iron(II) (as FeCl_2_) and 10% (vol/vol) of culture HP of the previous transfer was inoculated (transfer 1). After 7 days, culture HP was transferred into fresh media, which was repeated two times (transfers 2 and 3). The supplements were always added a minimum of 1 day in advance, due to precipitation of iron(II)-phosphate minerals (i.e., vivianite) with phosphate stemming from the growth media (26).

To verify that culture HP is not using the traces of OC present in the MQ water, the NRFeOx culture was spiked with 2 mM iron(II) after one transfer (7 days) because any residual OC stemming from the water should be used up during the first transfer already (experimental setup illustrated in Fig. S10a). To account for nitrate reduction and cell growth due to usage of internally stored OC, culture HP was transferred onto growth media supplemented with only 1 mM nitrate (as KNO_3_) (experimental setup illustrated in Fig. S10b).

For experiments testing arsenite toxicity effects and potential usage as an electron donor, 100 µM As(III) (as NaAsO_2_) was supplemented to culture HP (experimental setup illustrated in Fig. S11). Here, the media was supplemented with 0.5 mM nitrate and 2 mM iron(II), lowering the ratio of nitrate to iron from 1:2 to 0.5:2.

To test optimum growth conditions of the NRFeOx culture, culture HP was incubated at standard conditions (growth media, 1 mM nitrate, 2 mM iron(II), pH 7) at different iron(II) concentrations (1, 2, 3, 4, and 5 mM iron(II); experimental setup illustrated in Fig. S12) and at different pH values (6.5, 6.8, 6.95, and 7.05) (experimental setup illustrated in Fig. S13).

### Geochemical analyses

To determine iron redox speciation, 100 µL of a sample was fixed in 400 µL of a mixture of 1 M HCl/40 mM sulfamic acid and quantified using a modified ferrozine assay for nitrite-containing samples (83, 84). Briefly, 20 µL of the sample is mixed with 80 µL of 1 M HCl or with 80 µL of hydroxylamine hydrochloride followed by incubation for 30 min to quantify iron(II) and iron(tot), respectively. Afterwards, 100 µL of ferrozine solution is added, well mixed, and incubated for 5 min prior to spectrophotometric detection at 562 nm (Thermo Scientific Multiskan Go Microplate Spectrophotometer). Ferrozine measurements were conducted in technical triplicates.

To determine nitrogen species, 200 µL of the sample was diluted in 800 µL of MQ water for nitrate and nitrite and 50 µL of the sample was diluted in 950 µL of MQ water for ammonium prior to analysis by a segmented flow analyzer (AutoAnalyzer3, SEAL Analytical, Germany), which is equipped with a dialysis membrane for removal of iron to prevent side reactions during analysis.

The pH was measured in the culture suspension using a benchtop pH meter (SG2, Mettler-Toledo GmbH, Germany) equipped with a pH electrode (InLab Easy DIN, Mettler-Toledo GmbH, Germany).

### Gas measurements

For gas measurements, 0.6 mL of headspace (total headspace volume between 25 and 30.4 mL) was withdrawn before liquid sampling and injected into helium-flushed headspace vials (total volume 12 mL). For the pH range experiment, nitrogen-flushed headspace vials were used. N_2_O was quantified with a custom-built gas chromatograph (TRACEGC 1300, ThermoFisher Scientific, modified by S + HA analytics) on a pulsed discharge detector. The sample was split into two columns, one for N_2_O with the following configurations: 30 m long, 0.53 mm ID TGBondQ column; 30 m long 0.25 mm ID TGBondQ+ column (ThermoFisher Scientific). Calibration was performed between 1 and 200 ppm N_2_O, with helium or nitrogen background, depending on the experiment. Since samples were only taken from the headspace, dissolved N_2_O concentrations were calculated using the Henry constant (41.12 atm M^−1^), thus accounting for equilibrium partitioning between liquid and gaseous phases. For calculating the amount of N_2_O-N stemming from reduced NO_3_^-^-N, both the gaseous (measured) and dissolved (calculated) N_2_O concentrations were considered.

### 16S rRNA gene quantification, amplicon sequencing, and evolutionary analysis

After 11 transfers under lithoautotrophic conditions, two Hungate Tubes of culture HP were selected for analysis of the microbial community composition by 16S rRNA amplicon sequencing. For DNA extraction, 2 mL of sample was taken from culture HP and centrifuged (20,238 *g*, 5 min). The supernatant was discarded and the pellet was immediately frozen and stored at −20°C. The Power Soil DNA Kit was used for DNA extraction following standard procedures provided by the manufacturer (Qiagen, Germany). DNA was eluted in 50 µL DNase-free water and stored at −20°C. Quantitative polymerase chain reaction (qPCR) was performed for bacterial 16S rRNA genes using primers 341F (85) and 797R (86) in technical triplicates using SybrGreen Supermix (5 µL per qPCR reaction, Bio-Rad Laboratories GmbH, Munich, Germany) on a C1000 Touch thermal cycler (CFX96TM real-time system). As standards, plasmid vectors (pCR2.1, Invitrogen, Darmstadt, Germany) containing a cloned 16S rRNA gene fragment from *Thiomonas* sp. were used. A sevenfold standard dilution series and a negative control (RNase-free water) were included in each qPCR assay. Standard concentrations were quantified using Qubit (Life Technologies, Carlsbad, CA, USA). Finally, data analysis was performed using the Bio-Rad CFX Maestro 1.1, software, version 4.1 (Bio-Rad, 2017). For 16S rRNA gene amplicon sequencing, the 16S rRNA gene was amplified using primers 515F and 806R (87) targeting the V4 region. Detailed information on the primers, primer sequence, and thermal program used for qPCR and polymerase chain reaction (PCR) is listed in Table S11. Library preparation steps (Nextera, Illumina) and 250 bp paired-end sequencing with MiSeq (Illumina, San Diego, CA, USA) using v2 chemistry were performed by Microsynth AG (Balgach, Switzerland). 95,453 and 97,902 read pairs were obtained for two samples. Sequencing data were analyzed with nf-core/ampliseq v2.3.1, which includes all analysis steps and software and is publicly available (88, 89), with Nextflow v21.10.3 (90) and singularity v3.8.7 (91). Primers were trimmed, and untrimmed sequences were discarded (4% per sample) with Cutadapt version 3.4 (92). Adapter and primer-free sequences were processed with DADA2 v1.22.0 (93) to eliminate PhiX contamination, trim reads (before median quality drops below 35; forward reads were trimmed at 181 bp and reverse reads at 167 bp), correct errors, merge read pairs, and remove PCR chimeras; ultimately, 34 ASVs were obtained across both samples and 18 ASVs representing >99.9% abundance were found in both samples. Taxonomic classification was performed with DADA2 and the SILVA v138 database (94).

The evolutionary history was inferred using the Maximum Likelihood method and the Tamura-Nei model (95). The tree with the highest log likelihood (−462.61) is shown in Fig. S1. The percentage of trees in which the associated taxa clustered together is shown next to the branches. Initial tree(s) for the heuristic search were obtained automatically by applying Neighbor-Join and BioNJ algorithms to a matrix of pairwise distances estimated using the Tamura-Nei model and then selecting the topology with superior log likelihood value. The tree is drawn to scale, with branch lengths measured in the number of substitutions per site. This analysis involved 13 nucleotide sequences. All positions containing gaps and missing data were eliminated (complete deletion option). The final data set contained a total of 251 positions. Evolutionary analyses were conducted in MEGA X (96).

### Cell-mineral interaction analysis by fluorescence and electron microscopy

Cell-mineral interactions were visually characterized after mixing 1 µL of stain (LIVE/DEAD BacLight Bacterial Viability and Counting Kit, molecular probes, ThermoFisher Scientific, Waltham, MA, USA) and 6 µL of culture sample using combined fluorescence and transmission light microscopy (Leica DM5500 B microscope, Leica HCX PL S-APO 40×/0.75 objective, Leica DFC360 FX camera, Leica Microsystems LAS AF software). Additional samples were prepared for scanning electron microscopy (Zeiss Crossbeam 550L Focused Ion Beam SEM). For this, 1.11 mL of 25% electron microscopy-grade glutaraldehyde was added to sealable tubes containing 10 mL of culture HP. Samples were incubated overnight at 5°C to allow for thorough fixation. To rinse cells of any unreacted glutaraldehyde after incubation, samples were centrifuged (5 min, 7,000 *g*) to form a bacterial pellet. The supernatant was removed and 1 mL of DI water was added to each tube. The tubes were vortexed, to evenly distribute the cells in solution, and incubated at room temperature (ca. 21°C) for 15 min. After incubation, the tubes were re-centrifuged (1 min, 7,000 *g*) and the supernatant was removed. This procedure was repeated using 25%, 50%, and 75% ethanol to dehydrate the sample (i.e., replace water with an organic solvent for drying). A 25 µL aliquots of sample was pipetted onto separate poly-L-lysine coated glass slides, which were placed at the bottom of a well-plate. A lid was placed on top of the well-plate to prevent evaporation and the samples were incubated for 30 min allowing cells and minerals to settle onto the poly-L-lysine. A 1 mL volume of 100% ethanol was added to the well to completely submerse the glass slide, which was incubated for an additional 15 min with the lid on. After incubation, the ethanol was removed and replaced with “fresh” 100% ethanol with a 15-min incubation. This procedure was repeated a third time using 100% ethanol, once with 250 µL of 100% ethanol and 250 µL of hexamethyldisilazane (HMDS, Sigma-Aldrich, St. Louis, MO, USA) and finally with 100% HMDS. The lid of the well-plate was placed slightly ajar, allowing the HMDS to evaporate overnight. Once dry, the glass slides were attached to an aluminum stub using carbon adhesive tabs. The prepared samples were coated with a 10-nm-thick deposition of gold to prevent charging during analysis. Micrographs were taken in secondary electron (SE) mode using the SEM operating with an acceleration voltage of 2.0 kV. To identify the elemental composition of mineral precipitates, spot analysis of energy dispersive spectroscopy (EDS) was obtained from representative samples using an Oxford Instruments EDS detector and the SEM operating at 20 kV. Spectral data were analyzed using AZtec.

### Mineral analysis

For Moessbauer spectroscopy analysis, 10 mL of suspension was filtered anoxically (0.45 µm, Millipore) after the third transfer from a randomly selected bottle. The filter was sealed between two layers of Kapton tape and stored under anoxic conditions at −20°C until analysis. Samples were inserted into a closed-cycle exchange gas cryostat (Janis cryogenics) under a helium gas flow to minimize air exposure. Transmission spectra were collected at 77 K and 5 K using a constant acceleration drive system (WissEL) in transmission mode with a ^57^Co/Rh source. All spectra were calibrated against a 7 µm thick α-^57^Fe foil that was measured at 295 K. Recoil (University of Ottawa) and the Voigt Based Fitting (VBF) routine (97) was applied for sample analysis. The half-width at half maximum was constrained to 0.134 mm s^−1^ during fitting.

### Data analysis

A non-parametric Wilcoxon signed-rank test for paired samples, with the Benjamini-Hochberg *P*-adjustment method, was applied using R (4.3.3) and its interface RStudio (2023.12.1+402) to identify differences in 16S rRNA gene copy numbers between day 0 and day 7. A non-parametric Kruskal-Wallis test combined with a Wilcoxon rank-sum test, with the Benjamini-Hochberg *P*-adjustment method, was applied to estimate differences in N_2_O production between treatments.

## Data Availability

Raw sequencing data have been deposited at NCBI in the Sequence Read Archive (SRA) under BioProject accession number PRJNA1123617. The data set supporting the findings of this study can be accessed on Zenodo via https://doi.org/10.5281/zenodo.13683983.

## AFTER EPUB

*[This article was published on 6 December 2024 with an error in Acknowledgments. The error was corrected in the current version, posted on 16 December 2024.]*

## SUPPLEMENTAL MATERIAL

Supplemental material aem.01262-24-s0001.pdf Figures S1 to S11; Tables S1 to S13.

ASM does not own the copyrights to Supplemental Material that may be linked to, or accessed through, an article. The authors have granted ASM a non-exclusive, world-wide license to publish the Supplemental Material files. Please contact the corresponding author directly for reuse.
