# Supplementary material for: Nitrous oxide is the main product during nitrate reduction by a novel lithoautotrophic iron(II)-oxidizing culture from an organic-rich paddy soil
Source: Appl Environ Microbiol. 2024 Dec 6;91(1):e01262-24. doi: 10.1128/aem.01262-24 (PMC11784278; doi:10.1128/aem.01262-24)
Supplement: Supplemental material — Figures S1 to S11; Tables S1 to S13. [file aem.01262-24-s0001.pdf]

Appendix of

Nitrous oxide is the main product during nitrate reduction by a novel lithoautotrophic iron(II)-oxidizing culture from an organic-rich paddy soil

Hanna Grimm[1], Jennifer Lorenz[1], Daniel Straub[2], Prachi Joshi[1], Jeremiah Shuster[1,3*], Christiane Zarfl[4], E. Marie Muehe[5,6], Andreas Kappler[1,7#]

[1]Geomicrobiology, Department of Geosciences, University of Tübingen, Tübingen, Germany

[2]Quantitative Biology Center (QBiC), University of Tübingen, Germany

[3]Tübingen Structural Microscopy Core Facility, University of Tübingen, Tübingen, Germany

[4]Environmental Systems Analysis, Department of Geosciences, University of Tübingen, Tübingen, Germany

[5]Plant Biogeochemistry, Department of Applied Microbial Ecology, Helmholtz Centre for Environmental Research - UFZ, Leipzig, Germany

[6]Plant Biogeochemistry, Department of Geosciences, University of Tübingen, Tübingen, Germany

[7]Cluster of Excellence: EXC 2124: Controlling Microbes to Fight Infection, Tübingen, Germany

*Present address: Jeremiah Shuster, Department of Earth Sciences, Western University, London, Canada

[#]Address correspondence to Andreas Kappler, andreas.kappler@uni-tuebingen.de

Appendix: Soil characterization

Basic soil properties were analyzed in triplicates on soil samples after removal of plant debris and larger gravel. For soil texture analysis, a soil dispersion was prepared by adding 25 mL of sodium pyrophosphate to 30 g of non-sieved, fresh soil. After 30 min of stirring, the soil dispersion was filled up to a volume of 1000 mL and analyzed with a PARIO Soil Particle Analyzer (Meter Group, Germany) (1). After analysis, the soil was sieved (2 mm, 630 µm, 200 µm and 63 µm) to determine the sand and the fine fraction. Total loss of soil sample after sieving was below 5%. To determine the particle density, 20 g of dry soil was weighed into capillary pycnometer, filled up with deionized water and stepwise degassed before weight determination (2). Water content was determined by drying paddy soil at 105°C for 72 h. Soil pH was determined by adding 2.5 mL MQ water to 1 g of soil sample (2.5:1, solution:soil) and measuring after 2 h and 24 h using a benchtop pH meter (SG2, Mettler-Toledo GmbH, Germany) equipped with a pH electrode (InLab Easy DIN, Mettler-Toledo GmbH, Germany) (3).The cation exchange capacity of the paddy soil was quantified with a 0.1 M $BaCl_2$ extraction (4 h) by microwave plasma atomic emission spectroscopy (4200 MP-AES, Agilent technologies, United States) (4). X-ray fluorescence was used to determine total elemental content of the paddy soil. Glass beads were prepared by mixing 0.2333 g of dried and mortared sample with 3.9666 g of Fluxana FX-X65 (lithium tetraborate:lithium metaborate 66%:34%) and melted in a platinum crucible using a Spetec Roto-Melt 2,0 µP at 0.45 for 6 min. Afterwards, the sample was poured into a platinum mold and loaded into a S8 Tiger (Bruker) prior to analysis. Total element concentrations were quantified using the calibration package GeoQuant (Bruker). The loss on ignition (LOI) was calculated as the percental weight difference between the dried (105°C for 24 h) and annealed sample powder (3000°C for

3 h) and is 6.06 wt%. The LOI and the total XRF sums add up to 99.93 wt%. Total soil carbon and nitrogen contents were analyzed for dry and mortared paddy soil samples by dry combustion (soliTOC cube, Elementar Analysensysteme GmbH, Germany). Water-extractable organic carbon and nitrogen species were determined after extraction of 1 g dry weight soil with 5 mL of MQ by an elemental analyzer (multi N/C, 2100S, Analytik Jena GmbH) and segmented flow analysis (CFA, AutoAnalyzer 3, SEAL Analytical, Germany), respectively. To evaluate the presence of different iron mineral phases and associated arsenic, sequential extractions were performed under anoxic conditions. Soil samples were extracted for 24 h with 1 M sodium acetate (pH 5, adjusted with acetic acid) targeting adsorbed iron(II) and iron in amorphous sulfide minerals (referred to as adsorbed Fe) (5, 6). It is known that sodium acetate also extracts carbonates (7), yet this is considered to play a minor role due to low total inorganic carbon contents and low pH (Table A9). This was followed by 2 h extraction with 0.5 M HCl, extracting poorly crystalline iron minerals and reduced iron(II) minerals such as $FeCO_3$ and FeS (referred to as poorly crystalline Fe) (8). Lastly, samples were extracted for 24 h with 6 M HCl for extraction of more crystalline iron mineral phases and poorly reactive sheet silicate iron or FeS species (referred to as crystalline Fe) (9).

Appendix: Tables

Table A1. Composition of growth media for lithoautotrophic nitrate-reducing, iron(II)-oxidizing microorganisms.

Chemical	Concentration mg L^{-1}	Molar mass g mol^{-1}	Concentration mM	Reference
KH_2PO_4	0.14	136.09	1.03	
NaCl	0.20	58.44	3.42	
NH_4Cl	0.30	53.49	5.61	
$MgSO_4 \cdot 7\ H_2O$	0.50	246.47	2.03	
$CaCl_2 \cdot 2\ H_2O$	0.10	147.01	0.68	
$NaHCO_3$	1.85	84.01	22.02	
Trace elements SL10	1.00			(10)
7 vitamine solution	1.00			(11)
Selenite/tungstate solution	0.10			(12)

Table A2. Attempts to isolate the relevant nitrate-reducing, iron(II)-oxidizing strain in the NRFeOx culture.

Isolation attempts	Condition	Description	Success
Growth media with nitrate+acetate	anoxic	Backtransfer to liquid growth medium containing nitrate+iron(II)	no
LB plates	oxic	Picking of single colonies with repetitive streaking on LB plates, backtransfer to liquid growth medium containing nitrate+iron(II)	no
	microoxic		no
Plates with growth media with nitrate+iron(II)	anoxic	picking of single colonies with repetitive streaking on LB plates, backtransfer to liquid growth media containing nitrate+iron(II)	no
Gradient tubes	microoxic		no

Table A3. Relative 16S rRNA gene sequence abundance in native paddy soil from which lithoautotrophic NRFeOx culture was enriched.

Taxa	Relative abundance %
Gallionella	0.30
Comamonadaceae	1.46
Rhodocyclaceae	0.14
Holophagaceae	0.20

Table A4. Extent and stoichiometric ratio of reduced nitrate and oxidized iron(II), percentage of N_2O-N of total reduced NO_3^--N and start and end 16S rRNA gene copy numbers for different experiments. 'T' stands for transfer.

		**Nitrate reduced**	**Iron(II) oxidized***	**Ratio nitrate$_{red.}$:iron(II)$_{ox.}$**	**N_2O-N of total reduced NO_3^--N**
		mM	mM		%
Main experiment	T1	0.53 ± 0.12	2.29 ± 0.16	0.23 ± 0.05	72.29±19.38
	T2	0.45 ± 0.03	1.95 ± 0.20	0.23 ± 0.03	88.46±4.61
	T3	0.64 ± 0.13	1.72 ± 0.14	0.37 ± 0.08	62.38±16.00
Spike		0.38 ± 0.13	1.62 ± 0.19	0.23 ± 0.08	
No Fe(II) addition		0.05 ± 0.12	0		
Arsenite	Standard+As(III), T1	0.10 ± 0.05	0.51 ± 0.17	0.20 ± 0.12	39.78±19.92
	Standard+As(III), T2	0.15 ± 0.07	0.15 ± 0.18	1.02 ± 1.34	24.30±6.79
	Standard+As(III), T3	0.06 ± 0.03	0.22 ± 0.25	0.28 ± 0.34	25.66±14.36
Fe(II) concentrations	1 mM	0.12 ± 0.07	0.74 ± 0.27	0.16 ± 0.11	
	2 mM	0.46 ± 0.16	1.71 ± 0.03	0.27 ± 0.10	
	3 mM	0.41 ± 0.21	1.88 ± 0.90	0.22 ± 0.15	
	4 mM	0.12 ± 0.08	0.45 ± 0.29	0.27 ± 0.25	
	5 mM	0.08 ± 0.04	0.29 ± 0.35	0.29 ± 0.38	
pH range	pH 7.05	0.32 ± 0.03	1.41 ± 0.05	0.23 ± 0.02	82.02±9.65
	pH 6.95	0.28 ± 0.06	1.13 ± 0.14	0.25 ± 0.06	103.16±7.29
	pH 6.8	0.40 ± 0.02	1.66 ± 0.01	0.24 ± 0.01	85.32±17.36
	pH 6.5	0.34 ± 0.04	1.56 ± 0.09	0.22 ± 0.03	80.04±17.55

*considers retrieved iron and initially 90% iron(II) present

Table A5. 16S rRNA gene copy numbers as a measure for microbial growth at the beginning and end of a transfer or experiment. Log2FC displays the log 2-fold change of 16S rRNA gene copy numbers between day 0 and day 7. The results of a non-parametric Wilcoxon signed-rank test for paired samples, with the Benjamini-Hochberg p-adjustment method (FDR: false discovery rate), are summarized. 'T' stands for transfer.

		**Start (day 0)**	**End (day 7)**	**Log2FC**	**n**	**W**	**FDR (adjusted p-value)**	**Effect size**
		16S rRNA gene copy no. mL^{-1}						
Main experiment	T2	$3.98{\times}10^{3} \pm 1.58{\times}10^{3}$	$4.31{\times}10^{5} \pm 2.46{\times}10^{5}$	6.76	4	-1.15	0.12	0.54
	T3	$6.15{\times}10^{3} \pm 2.02{\times}10^{3}$	$1.23{\times}10^{6} \pm 6.28{\times}10^{5}$	7.64	4	-1.15	0.12	0.54
spike		$2.20{\times}10^{6} \pm 1.40{\times}10^{6}$	$1.29{\times}10^{6} \pm 6.09{\times}10^{5}$	-0.77	4	-1.15	0.12	0.54
no Fe(II) addition		$2.49{\times}10^{4} \pm 1.05{\times}10^{4}$	$1.52{\times}10^{6} \pm 1.45{\times}10^{6}$	5.93	3	-1.15	0.25	0.47
Arsenite	Standard+As(III), T1	$6.27{\times}10^{3} \pm 1.41{\times}10^{3}$	$5.68{\times}10^{4} \pm 1.44{\times}10^{4}$	3.18	3	-1.15	0.25	0.47
	Standard+As(III), T3	$6.74{\times}10^{3} \pm 8.00{\times}10^{2}$	$4.03{\times}10^{4} \pm 3.58{\times}10^{4}$	2.58	3	-1.15	0.25	0.47

Table A6. 16S rRNA gene copy numbers at day 0 (start) and day 7 (end) in different treatments. 1 mM nitrate, 2 mM Fe(II) reflect standard conditions and 1 mM nitrate setups the absence of an electron donor.

Day	16S rRNA gene copy no. mL^{-1}	
	1 mM nitrate, 2 mM Fe(II)	1 mM nitrate
0	1.32E+04 ± 9.48E+03	2.49E+04 ± 1.05E+04
7	1.33E+06 ± 6.95E+05	1.52E+06 ± 1.45E+06

Table A7. Moessbauer spectra hyperfine parameters for the mineral phase produced by the lithoautotrophic NRFeOx culture after 7 days of incubation (transfer 3).

Temperature	Phase	Iron mineral phase	CS	ΔE_Q	ε	B_{hf}	Pop	χ2
K			mm s^{-1}	mm s^{-1}	mm s^{-1}	T	%	
77	Db	Fe(III)	0.48	0.84			100	0.65
5	Sxt1	Fe(III), Fh	0.52		-0.05	42.03	57.1	0.76
	Sxt2	Fe(III), Fh	0.34		0.04	34.59	42.9	

Db: doublet, Sxt: sextet, Iron mineral phase - Fe(III): ferric Fe, Fh: ferrihydrite, CS: center shift, ΔE_Q: quadrupole splitting, ε: quadrupole shift, B_{hf}: hyperfine field, Pop: relative abundance, χ2: goodness of fit.

Table A8. pH values at day 0 (start) and day 7 (end) in different treatments during the pH range experiment.

Treatment	pH value	
	Day 0	Day 7
**pH 7.05**	7.06 ± 0.01	7.07 ± 0.00
**pH 6.95**	6.95 ± 0.01	6.97 ± 0.01
**pH 6.8**	6.82 ± 0.00	6.76 ± 0.01
**pH 6.5**	6.55 ± 0.04	6.47 ± 0.02
**Control pH 7.05**	7.09 ± 0.01	7.08 ± 0.01
**Control pH 6.5**	6.52 ± 0.03	6.49 ± 0.01

Table A9. Basic soil properties of paddy soil collected from Huilongpu Town, Hunan province, China.

		Huilongpu, China
**Coordinates**		28°12′16″ N, 112°26′32″ E
**Parent material**		River alluvium
**Paddy management**		rice-rice
**Soil texture**		
Sand	(%)	16.91 ± 0.08
Silt	(%)	60.00 ± 6.00
Clay	(%)	19.33 ± 5.51
**CEC**	($cmol\ kg^{-1}$)	14.09 ± 0.06
**pH_{MQ}**		7.31 ± 0.15
**Water content**	(%)	44.84 ± 1.33
**TOC**	($g\ kg^{-1}$)	35.47 ± 2.53
**TIC**	($g\ kg^{-1}$)	0.54 ± 0.37
**TN**	($g\ kg^{-1}$)	3.30 ± 0.31
**Water-extractable OC**	($g\ kg^{-1}$)	0.09 ± 0.02
**Water-extractable N**	($g\ kg^{-1}$)	0.02 ± 0.00
**Adsorbed Fe***	($g\ kg^{-1}$)	0.75 ± 0.12
**Poorly crystalline Fe***	($g\ kg^{-1}$)	1.94 ± 0.20
**Crystalline Fe***	($g\ kg^{-1}$)	11.64 ± 1.22
**Total extractable Fe***	($g\ kg^{-1}$)	14.34 ± 1.24
**Adsorbed Fe-As***	($mg\ kg^{-1}$)	0.00 ± 0.00
**Poorly crystalline Fe-As***	($mg\ kg^{-1}$)	1.15 ± 0.27
**Crystalline Fe-As***	($mg\ kg^{-1}$)	3.91 ± 0.33
**Total extractable Fe-As***	($mg\ kg^{-1}$)	5.06 ± 0.43
**Na°**	($g\ kg^{-1}$)	2.05
**Mg°**	($g\ kg^{-1}$)	2.94
**Al°**	($g\ kg^{-1}$)	57.91
**Si°**	($g\ kg^{-1}$)	323.93
**P°**	($g\ kg^{-1}$)	0.71
**S°**	($g\ kg^{-1}$)	0.87
**K°**	($g\ kg^{-1}$)	13.83
**Ca°**	($g\ kg^{-1}$)	6.27
**Ti°**	($g\ kg^{-1}$)	5.60
**V°**	($g\ kg^{-1}$)	0.06
**Cr°**	($g\ kg^{-1}$)	0.05
**Mn°**	($g\ kg^{-1}$)	0.22
**Fe°**	($g\ kg^{-1}$)	21.09
**Ni°**	($g\ kg^{-1}$)	0.13
**Zn°**	($g\ kg^{-1}$)	0.02
**Zr°**	($g\ kg^{-1}$)	0.08
**Ba°**	($g\ kg^{-1}$)	0.04

Average and standard deviation are represented by triplicate measurements
*Obtained by sequentially extracting paddy soil samples with 1 M Na-acetate (adsorbed), 0.5 M HCl (poorly crystalline) and 6 M HCl (crystalline)

°Obtained by XRF analysis

Table A10. Results of a) Kruskal-Wallis test and b) Wilcoxon rank-sum test with Benjamini-Hochberg-adjusted p-values (FDR, false discovery rate) to identify differences in N_2O production of different treatments.

a) Kruskal-Wallis test.

**Comparison**	χ^2	**df**	**p-value**
Treatment vs N_2O	18.966	5	0.001951

b) Wilcoxon rank-sum test, Benjamini-Hochberg adjusted p-values (FDR).

	**N_2O-N of total reduced NO_3^--N** %	**n**	**Comparison with**	**FDR (adjusted p-value)**
**Ratio N:Fe, 1:2 (Standard conditions)**	88.77 ± 26.57	15	Ratio N:Fe, 0.5:2	1
			Ratio N:Fe, 0.5:2 + As(III)	0.00044
			pH 7.05	1
			pH 6.8	1
			pH 6.5	1
**Ratio N:Fe, 0.5:2**	76.83 ± 29.14	9	Ratio N:Fe, 0.5:2 + As(III)	0.00123
			pH 7.05	1
			pH 6.8	1
			pH 6.5	1
**Ratio N:Fe, 1:0.5 + As(III)**	29.48 ± 25.48	9	pH 7.05	0.03409
			pH 6.8	0.03409
			pH 6.5	0.05455
**pH 7.05**	80.04 ± 17.55	3	pH 6.8	1
**pH 6.8**	85.32 ± 17.36	3	pH 6.5	1
**pH 6.5**	82.02 ± 9.65	3	pH 6.5	1

Table A11. Detailed information of qPCR and PCR analysis for 16S rRNA gene copy numbers and amplicon sequencing, respectively.

Target gene	Standard	Primer	Primer sequence (5' -> 3')	Primer concentration	Thermal program	References
				nM		
Bacterial 16S rRNA gene	*Thiomonas* sp.	341F	CCTACGGGAGG CAGCAG	250	95°C - 5'; (95°C - 10"; 60°C - 15") x 40; 95°C - 30"; 65-95°C - 5"	(13, 14)
		797R	GGACTACCAGG GTATCTAATCCT GTT	250		
Bacterial 16S rRNA gene amplicon sequencing	*Thiomonas* sp.	515F	TCGTCGGCAGC GTCAGATGTGT ATAAGAGACAG GTGYCAGCMGC CGCGGTA	250	94°C - 3'; (94°C - 30"; 55°C - 30";72°C - 30") x 25; 72°C - 8'; 4°C	(15)
		806R	GTCTCGTGGGC TCGGAGATGTG TATAAGAGACA GGGACTACNVG GGTWTCTAAT	250		

Appendix: Figures

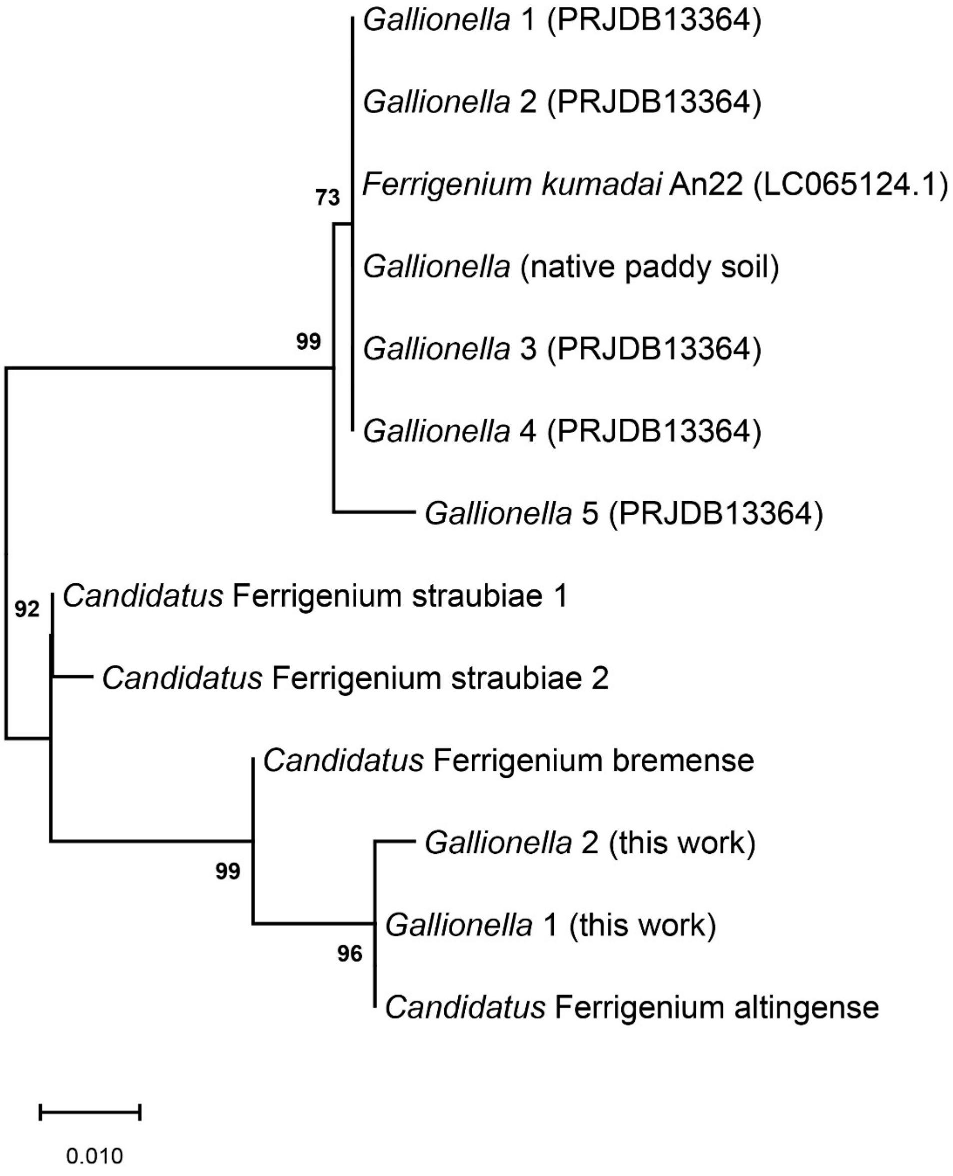

Figure A1. Maximum-likelihood phylogenetic tree of *Gallionella* species in lithoautotrophic nitrate-reducing, iron(II)-oxidizing enrichment cultures and paddy soils. In the enrichment culture obtained in this study, two *Gallionella* species were enriched; Gallionella 1 (71.39±0.3% relative abundance) and Gallionella 2 (2.69±0.01% relative abundance). The tree was constructed using the maximum-likelihood method based on 16S rRNA gene sequences with a total of 251 positions in the final dataset. The percentage of trees in which the associated taxa clustered together is shown next to the branches. The scale bar represents the number of substitutions per site.

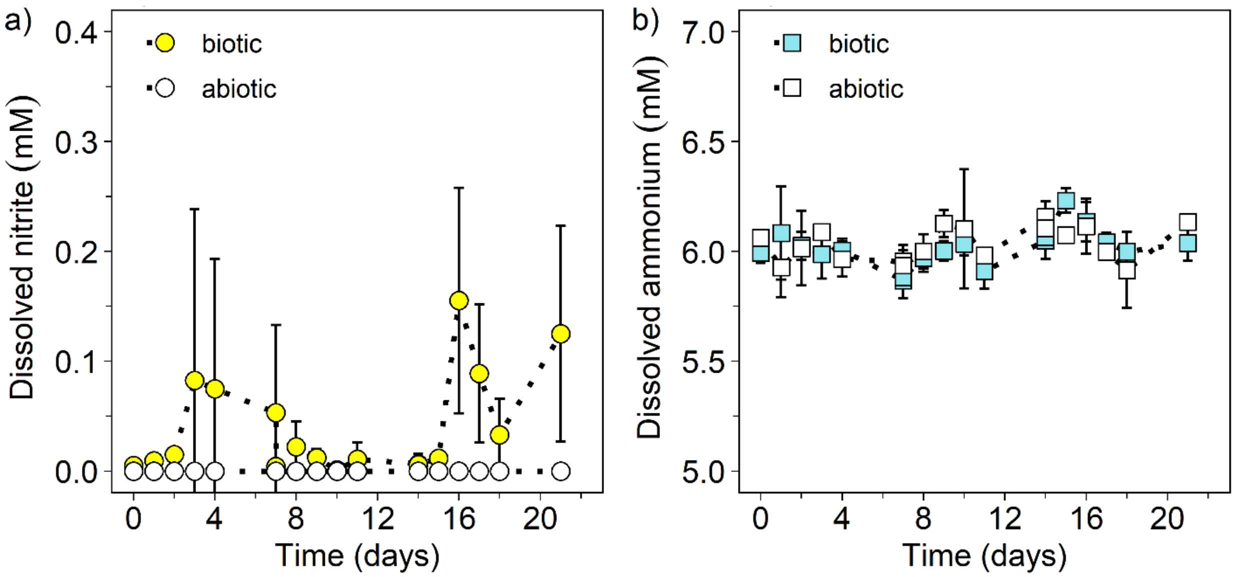

Figure A2. Nitrite (a) and ammonium (b) concentrations over three consecutive transfers each lasting 7 days (Transfer 1: 0-7 days, Transfer 2: 7-14 days and Transfer 3: 14-21 days. Nitrite (a) is displayed as yellow circles and ammonium (b) as blue squares, abiotic treatments in white. Note that the y-axis in b) ranges from 5 to 7 mM. Mean ± standard deviation is shown of four replicates.

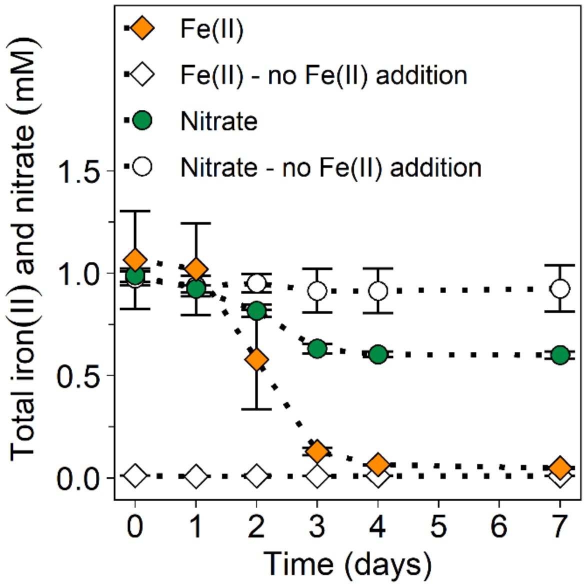

Figure A3. Total iron(II) (diamonds) and nitrate (circles) concentrations over 7 days in biotic treatments. Treatments with supplemented Fe(II) are represented as colored symbols (ratio N:Fe=1:2, pH 7), treatments without Fe(II) addition in white (ratio N:Fe=1:0, pH 7). Mean ± standard deviation is shown of three replicates.

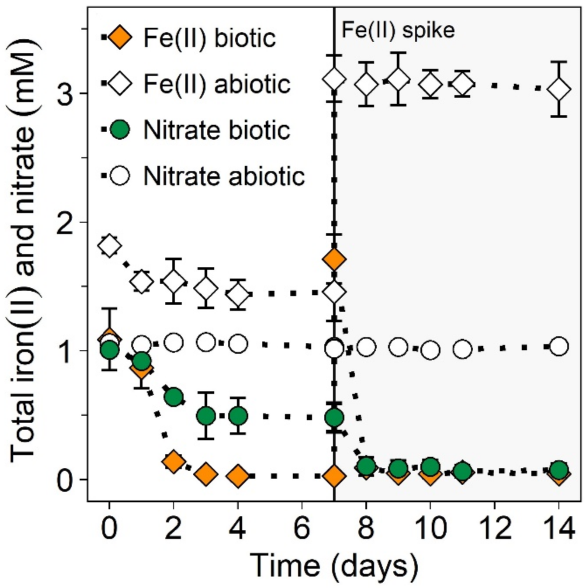

Figure A4. Total iron(II) (diamond) and nitrate (circle) concentrations over 14 days. 2 mM of Fe(II) was spiked after 7 days, visually separated by lines and color. Biotic treatments are represented as colored symbols, abiotic treatments in white. Mean ± standard deviation is shown of four replicates.

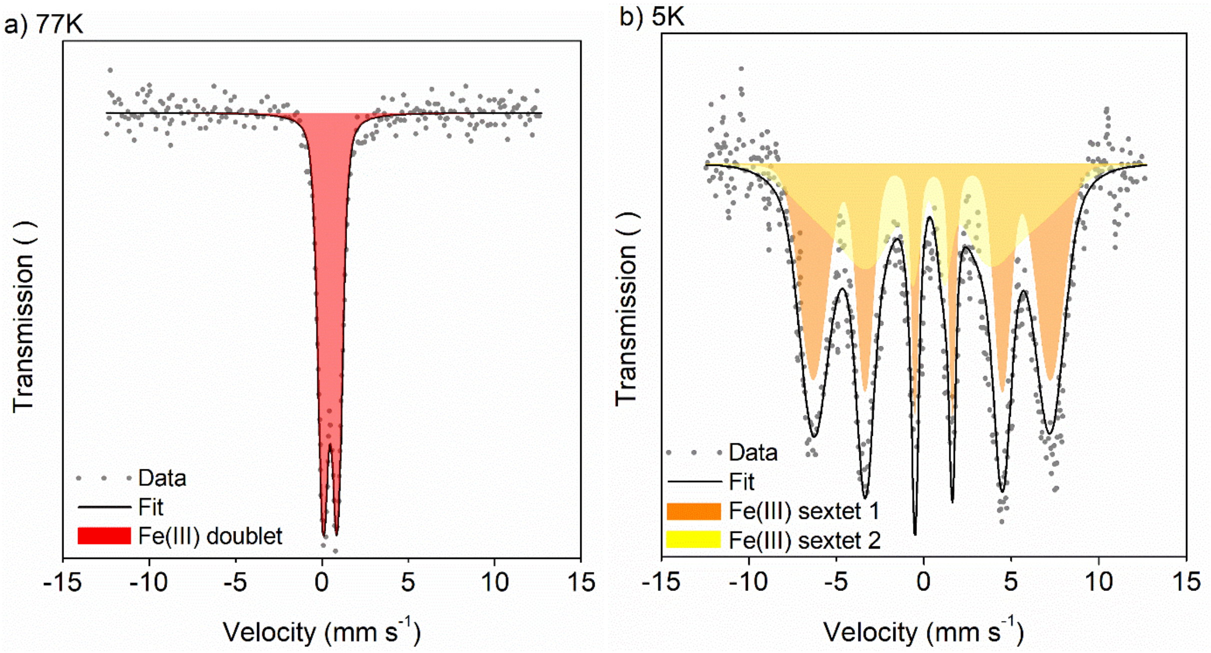

Figure A5. Moessbauer spectra collected at 77 K (a) and 5 K (b) of the mineral phase formed by the lithoautotrophic NRFeOx enrichment culture after 7 days of incubation. The spectra illustrates the data (grey dots), the fitted data (black solid line) and the different mineral phases (iron(III) doublet: red, iron(III) sextet 1: orange, iron(III) sextet 2: yellow). Grey dots represent data points, black solid lines the fitted data, and the colored areas the fitted mineral phases. Hyperfine parameters can be taken from Table A7.

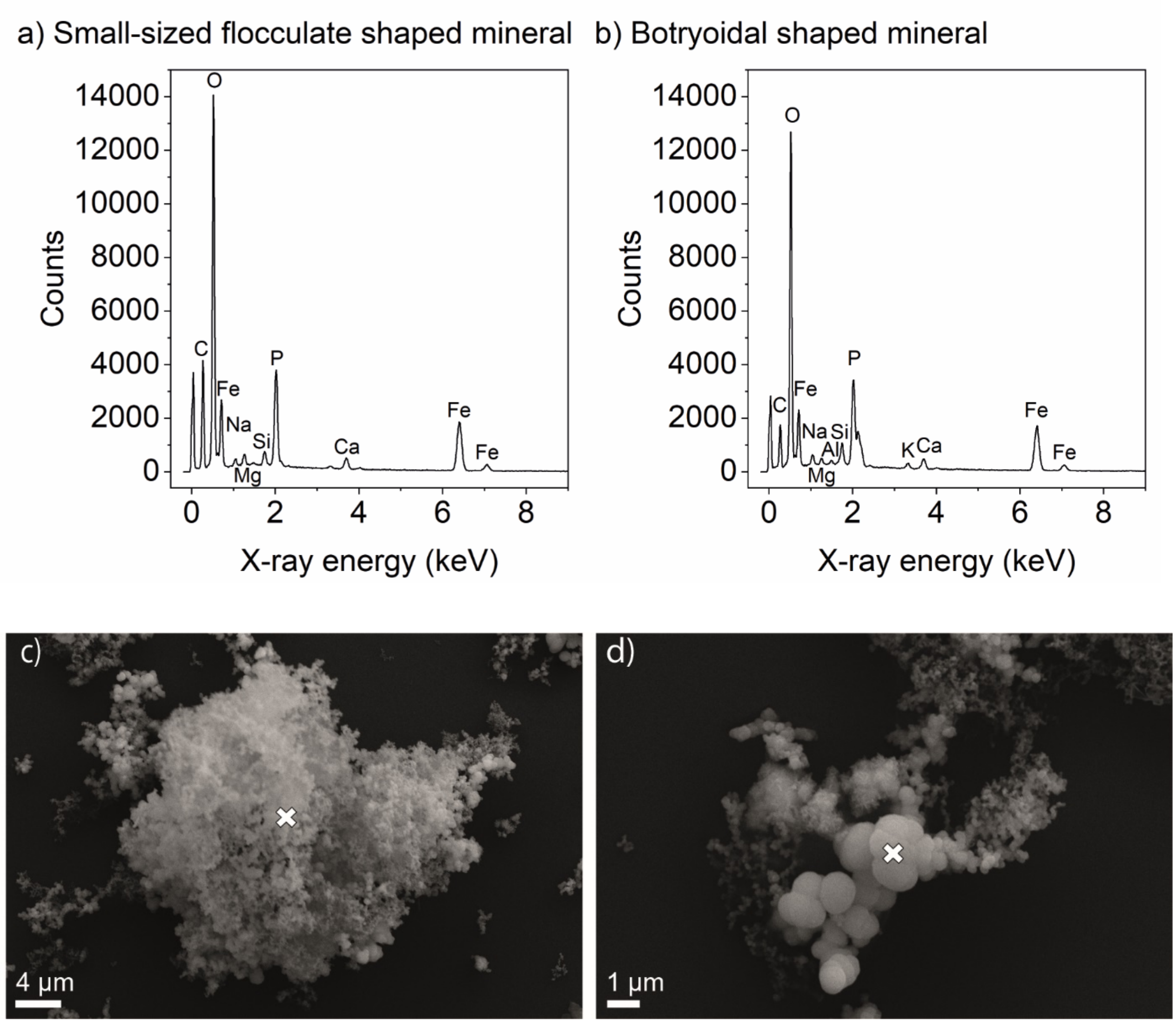

Figure A6. EDS spectra collected from a small-sized flocculate shaped mineral (a) and a botryoidal shaped mineral (b) from the lithoautotrophic NRFeOx culture after 7 days (Transfer 3). Corresponding SEM pictures of collected EDS spectra of a small-sized flocculate shaped mineral (c) and a botryoidal shaped mineral (d). The cross indicates the point of measurement.

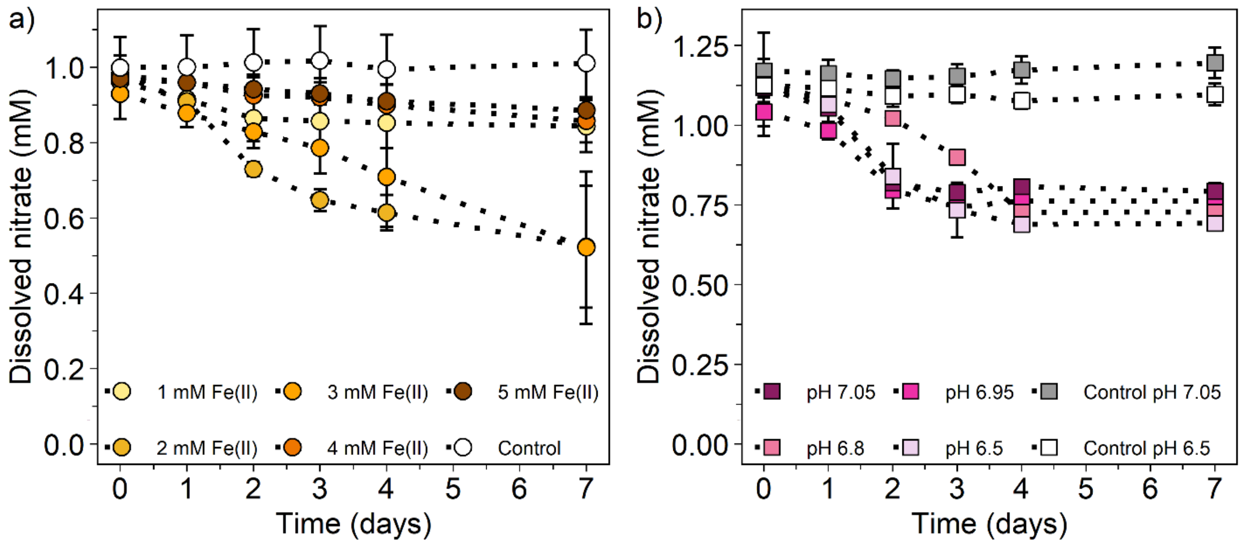

Figure A7. Nitrate concentrations (a) for cultures set up with different Fe(II) concentrations and (b) for cultures set up at different pH values over 7 days. Treatments in a) are showing different iron concentrations as circles with darker colors representing higher iron concentrations. The control (white) represents abiotic conditions using 5 mM iron(II) and 1 mM nitrate. Treatments in b) are displaying different pH values as squares with darker colors representing higher pH values. The controls represent abiotic conditions at pH 6.50 (white) and pH 7.05 (grey) using 2 mM iron(II) and 1 mM nitrate. Mean ± standard deviation is shown of three replicates.

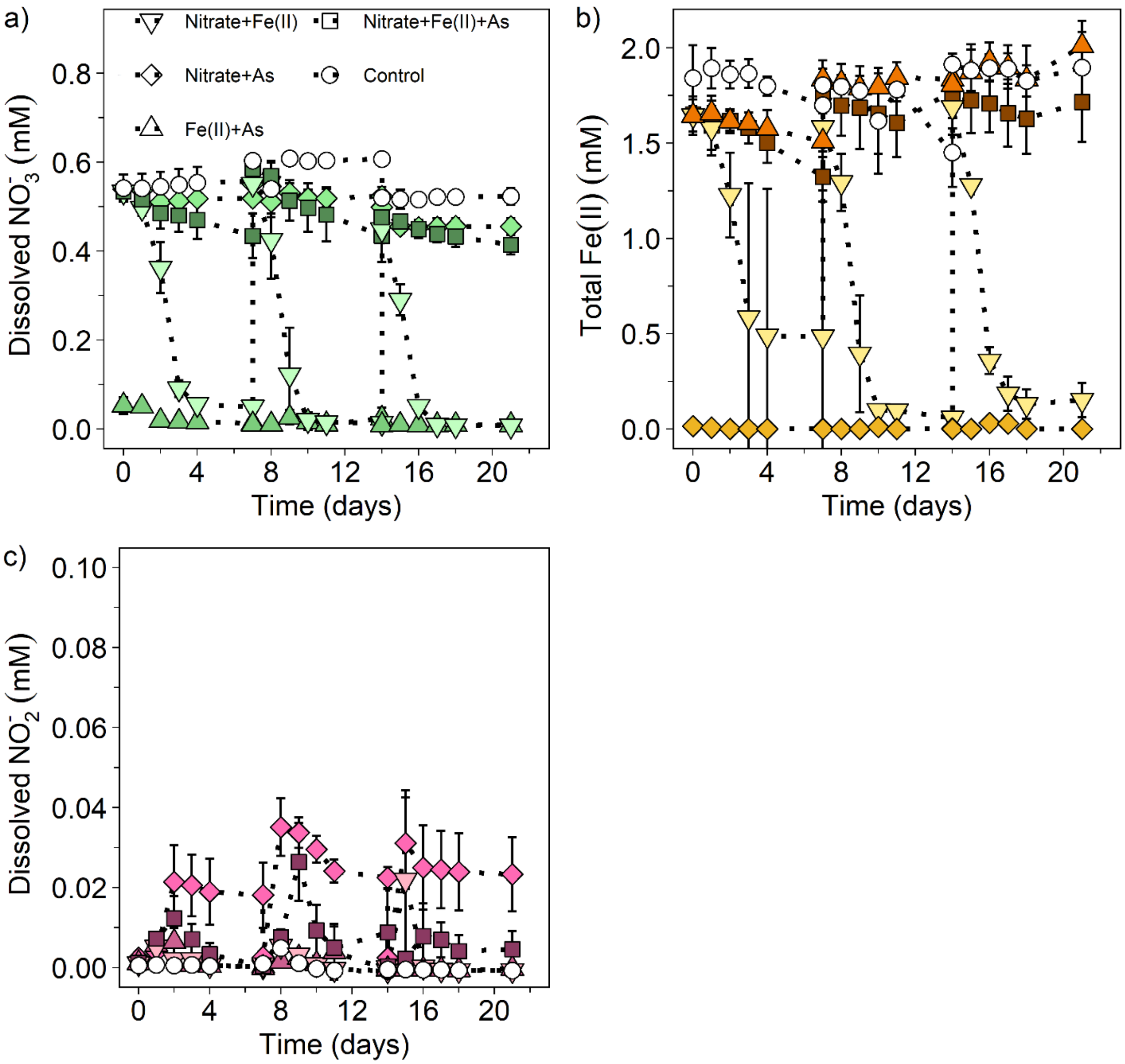

Figure A8. Dissolved nitrate (a), total Fe(II) (b) and dissolved nitrite (c) concentrations over three consecutive transfers (each 7 days) for four different biotic setups (upside down triangle: 0.5 mM nitrate, 2 mM Fe(II); diamond: 0.5 mM nitrate, 100 µM arsenite); triangle: 2 mM Fe(II), 100 µM arsenite; square: 0.5 mM nitrate, 2 mM Fe(II), 100 µM arsenite) and one abiotic control (circle). Mean ± standard deviation is shown of three replicates.

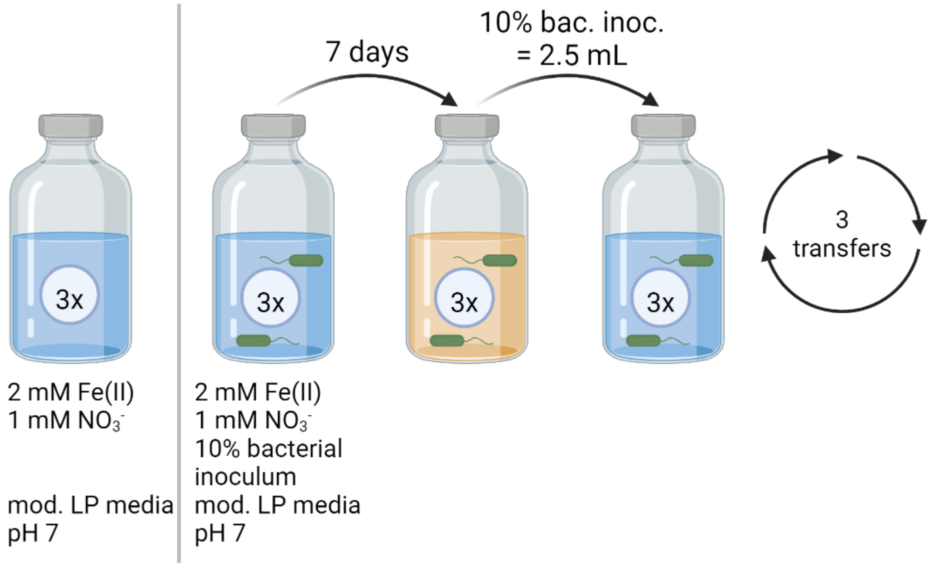

Figure A9. Experimental setup for growing the NRFeOx culture under autotrophic conditions over three consecutive transfers. Grey line separates and provides information of the media composition of abiotic (left) and biotic (right) setups.

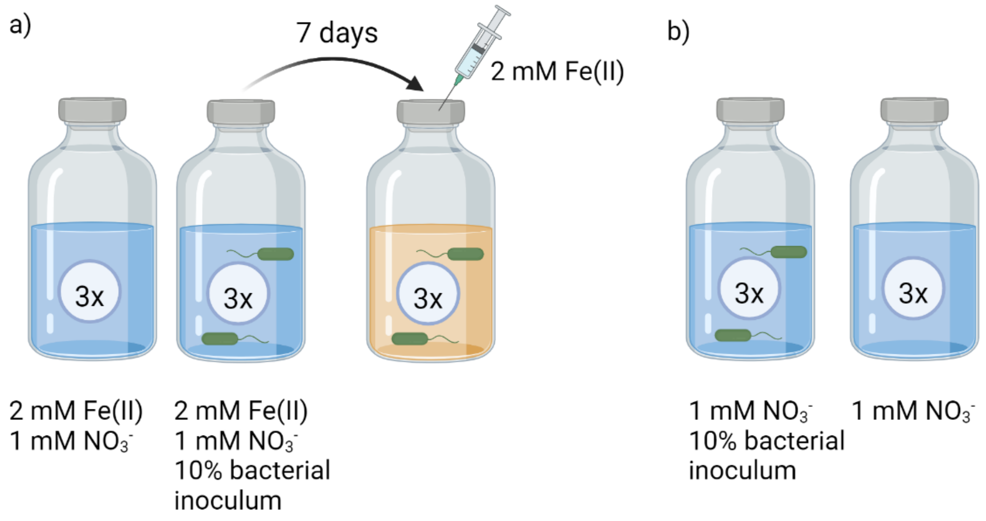

Figure A10. Experimental setup for examining the use of residual OC stemming from the MQ water (a) and of internally stored OC (b) by the NRFeOx culture.

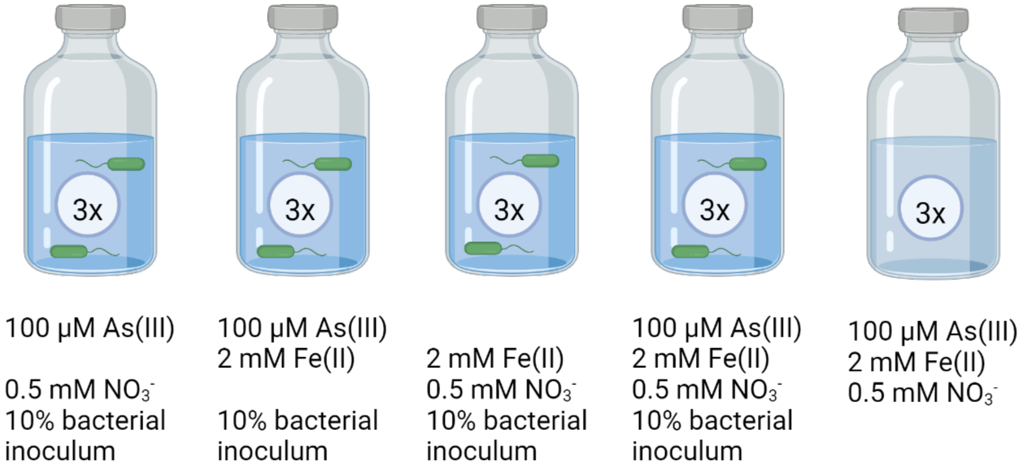

Figure A11. Experimental setup for testing arsenite toxicity and the potential of arsenite as electron donor for the lithoautotrophic NRFeOx culture.

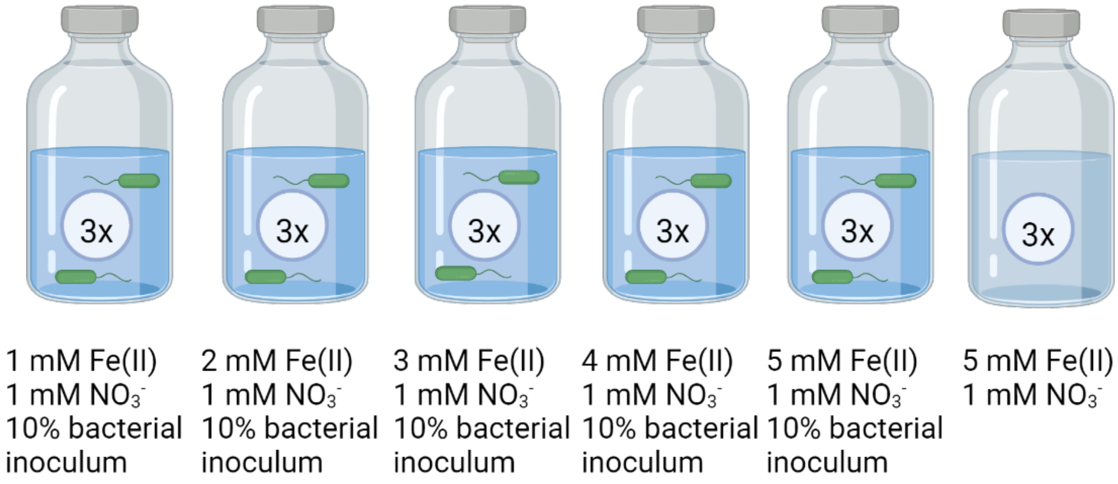

Figure A12. Experimental setup for using different concentrations of Fe(II) by the lithoautotrophic NRFeOx culture.

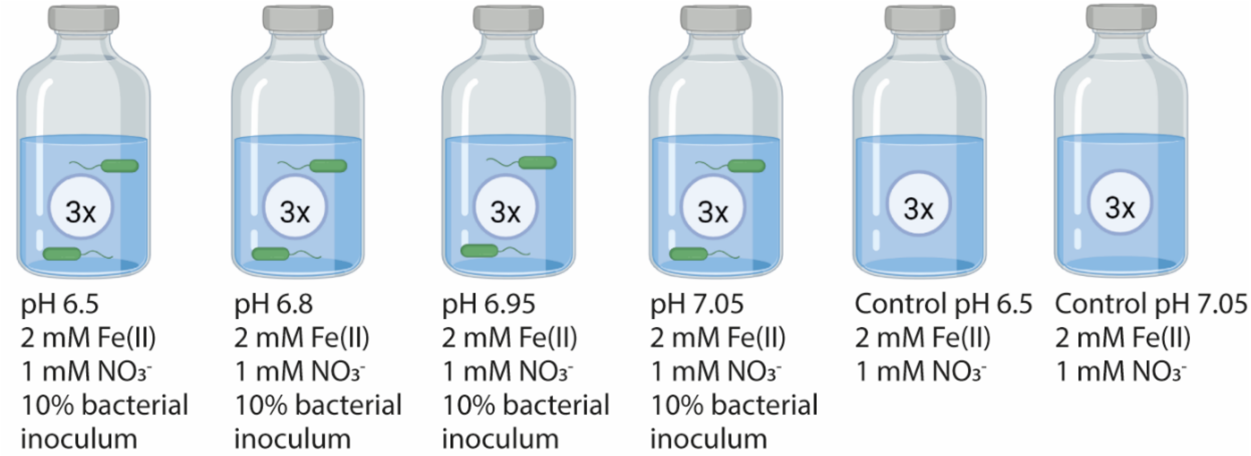

Figure A13. Experimental setup for testing different pH values for cultivation of the lithoautotrophic NRFeOx culture.

References

1. DIN 18123. 2011. Soil, investigation and testing - Determination of grain-size distribution.

2. DIN 18124. 2011. Soil, investigation and testing - Determination of density of solid particles - Capillary pyknometer, wide mouth pycnometer, gas pycnometer.

3. ISO 10390. 2021. Soil, treated biowaste and sludge - Determination of pH.

4. Muehe EM, Wang T, Kerl CF, Planer-Friedrich B, Fendorf S. 2019. Rice production threatened by coupled stresses of climate and soil arsenic. Nature communications 10:4985.

5. Roden EE, Zachara JM. 1996. Microbial Reduction of Crystalline Iron(III) Oxides: Influence of Oxide Surface Area and Potential for Cell Growth. Environ Sci Technol 30:1618–1628.

6. Shannon RD, White JR. 1991. The selectivity of a sequential extraction procedure for the determination of iron oxyhydroxides andiron sulfides in lake sediments. Biogeochemistry 14:193–208.

7. Tessier A, Campbell PGC, Bisson M. 1979. Sequential extraction procedure for the speciation of particulate trace metals. Anal Chem 51:844–851.

8. Heron G, Crouzet C, Bourg AC, Christensen TH. 1994. Speciation of Fe(II) and Fe(III) in Contaminated Aquifer Sediments Using Chemical Extraction Techniques. Environ Sci Technol 28:1698–1705.

9. Lueder U, Maisch M, Laufer K, Jørgensen BB, Kappler A, Schmidt C. 2020. Influence of Physical Perturbation on Fe(II) Supply in Coastal Marine Sediments. Environ Sci Technol 54:3209–3218.

10. Widdel F, Kohring G-W, Mayer F. 1983. Studies on dissimilatory sulfate-reducing bacteria that decompose fatty acids: III. Characterization of the filamentous gliding Desulfonema limicola gen. nov. sp. nov., and Desulfonema magnum sp. nov. Arch Microbiol 134:286–294.

11. Widdel F, Pfennig N. 1981. Studies on dissimilatory sulfate-reducing bacteria that decompose fatty acids. I. Isolation of new sulfate-reducing bacteria enriched with acetate from saline environments. Description of Desulfobacter postgatei gen. nov., sp. nov. Arch Microbiol 129:395–400.

12. Jakus N, Blackwell N, Osenbrück K, Straub D, Byrne JM, Wang Z, Glöckler D, Elsner M, Lueders T, Grathwohl P, Kleindienst S, Kappler A. 2021. Nitrate Removal by a Novel Lithoautotrophic Nitrate-Reducing, Iron(II)-Oxidizing Culture Enriched from a Pyrite-Rich Limestone Aquifer. Applied and environmental microbiology 87:e0046021.

13. Muyzer G, De Waal EC, Uitterlinden AG. 1993. Profiling of complex microbial populations by denaturing gradient gel electrophoresis analysis of polymerase chain reaction-amplified genes coding for 16S rRNA. Appl Environ Microbiol 59:695–700.

14. Nadkarni MA, Martin FE, Jacques NA, Hunter N. 2002. Determination of bacterial load by real-time PCR using a broad-range (universal) probe and primers set. Microbiology 148:257–266.

15. Caporaso JG, Lauber CL, Walters WA, Berg-Lyons D, Lozupone CA, Turnbaugh PJ, Fierer N, Knight R. 2011. Global patterns of 16S rRNA diversity at a depth of millions of sequences per sample. PNAS 108 Suppl 1:4516–4522.
